# A crotonyl-CoA reductase-carboxylase independent pathway for assembly of unusual alkylmalonyl-CoA polyketide synthase extender units

**DOI:** 10.1038/ncomms13609

**Published:** 2016-12-21

**Authors:** Lauren Ray, Timothy R. Valentic, Takeshi Miyazawa, David M. Withall, Lijiang Song, Jacob C. Milligan, Hiroyuki Osada, Shunji Takahashi, Shiou-Chuan Tsai, Gregory L. Challis

**Affiliations:** 1 Department of Chemistry, University of Warwick, Coventry CV4 7AL, UK; 2 Departments of Molecular Biology and Biochemistry, Chemistry, and Pharmaceutical Sciences, University of California, Irvine, California 92697, USA; 3 Chemical Biology Research Group, RIKEN Center for Sustainable Resource Science, Saitama 351-0198, Japan; 4 Graduate School of Science and Engineering, Saitama University, Saitama 338-8570, Japan

## Abstract

Type I modular polyketide synthases assemble diverse bioactive natural products. Such multienzymes typically use malonyl and methylmalonyl-CoA building blocks for polyketide chain assembly. However, in several cases more exotic alkylmalonyl-CoA extender units are also known to be incorporated. In all examples studied to date, such unusual extender units are biosynthesized via reductive carboxylation of α, β-unsaturated thioesters catalysed by crotonyl-CoA reductase/carboxylase (CCRC) homologues. Here we show using a chemically-synthesized deuterium-labelled mechanistic probe, and heterologous gene expression experiments that the unusual alkylmalonyl-CoA extender units incorporated into the stambomycin family of polyketide antibiotics are assembled by direct carboxylation of medium chain acyl-CoA thioesters. X-ray crystal structures of the unusual β-subunit of the acyl-CoA carboxylase (YCC) responsible for this reaction, alone and in complex with hexanoyl-CoA, reveal the molecular basis for substrate recognition, inspiring the development of methodology for polyketide bio-orthogonal tagging via incorporation of 6-azidohexanoic acid and 8-nonynoic acid into novel stambomycin analogues.

## Abstract (web summary)

Polyketides are typically assembled from a starter unit and malonyl- and/or methylmalonyl-CoA-derived extender units, but the macrolide antibiotics stambomycins incorporate non-standard alkylmalonyl-CoA extender units. Here, the authors describe the biosynthetic pathway responsible for this unusual synthesis.

Modular polyketide synthases (PKSs) are bacterial molecular machines responsible for the assembly of numerous structurally-complex bioactive natural products with diverse applications in medicine and agriculture^1^. Important examples of modular PKS products include: erythromycin, rifamycin and fidaxomicin (antibacterials); amphotericin and nystatin (antifungals); rapamycin and FK-506 (immunosuppressants); spinosyns (insecticides) and avermectins (insecticides and anthelmintics). These remarkable multienzymes employ various tactics to generate structural diversity, such as variation in the number of modules within the assembly line, and of the α and β-carbon-modifying domains within each module; in *trans* recruitment of a range of enzymes that modify the growing polyketide during chain assembly; utilization of several distinct mechanisms for termination of chain assembly, and a wide variety of starter and extender units^1^.

The stambomycins **1**–**4** (Fig. 1a) are a novel class of macrolide antibiotics recently identified as products of a modular PKS via a rational activation of a silent biosynthetic gene cluster in *Streptomyces ambofaciens* ATCC23877 (refs 2, 3). The majority of the stambomycin PKS chain elongation modules are predicted to employ widely-used malonyl and (2*S*)-methylmalonyl-CoA extender units (Fig. 1)^2^. The exception is module 12, which is postulated to incorporate several atypical pentyl- and hexylmalonyl-CoA-based extender units, giving rise to the structural variants of the stambomycin complex (Fig. 1)^2^. Several unusual extender units are known to be utilized by other modular PKSs, including those responsible for the assembly of salinosporamide A (chloroethylmalonyl-CoA) (ref. 4), cinnabaramide A (*n*-hexylmalonyl-CoA) (ref. 5), filipin III (*n*-hexylmalonyl-CoA) (ref. 6), antimycins (*n*-butylmalonyl-CoA, *n*-hexylmalony-CoA) (ref. 7), splenocins (benzylmalonyl-CoA) (ref. 8), FK-506 (allylmalonyl-CoA) (ref. 9), chlorizidines (dichloropyrrolyl-propylmalonyl-ACP) (ref. 10), and the reveromycins (*n*-butylmalonyl-CoA, *iso*-butylmalonyl-CoA, *n*-pentylmalonyl-CoA and *n*-hexylmalonyl-CoA) (ref. 11). In all of these examples, the final step of unusual extender unit biosynthesis is catalysed by a homologue of crotonyl-CoA reductase/carboxylase (CCRC) and involves NADPH-mediated reduction of an α, β-unsaturated thioester, followed by trapping of the resulting enolate by reaction with carbon dioxide^12^,^13^,^14^. The genes encoding such CCRC homologues are invariably clustered with the genes encoding the polyketide synthases they supply^4^,^5^,^6^,^7^,^8^,^9^,^10^,^11^. Remarkably, no genes encoding CCRC homologues can be found in the stambomycin biosynthetic gene cluster (BGC) (ref. 2), suggesting that the unusual extender units incorporated into the stambomycins may be assembled by a CCRC-independent mechanism.

Acyl-CoA carboxylases (YCCs) generate the malonyl and methylmalonyl-CoA extender units commonly utilized by modular PKSs, in addition to carrying out a variety of other carboxylation reactions^15^. YCCs consist of three components: a biotin carboxylase (BC), a biotin carboxyl carrier protein (BCCP) and a carboxyl transferase (CT)^15^. The distrubtion of these components between protein subunits differs depending on the organism and the nature of the substrate^15^. In *Streptomyces* species the YCCs acetyl-CoA carboxylase (ACC) and propionyl-CoA carboxylase (PCC) assemble malonyl and methylmalonyl-CoA via carboxylation of acetyl- and propionyl-CoA, respectively^16^,^17^. ACC and PCC consist of a common α-subunit, AccA2 and dedicated β-subunits, AccB and PccB, respectively. AccA2 contains an N-terminal BC domain and a C-terminal BCCP domain and AccB/PccB contain CT domains with preference for acetyl-CoA/propionyl-CoA as substrates. In addition, a non-catalytic ɛ-subunit has been reported to enhance the catalytic activity of ACC and PCC *in vitro*^16^. The *samR0483* gene within the stambomycin biosynthetic gene cluster encodes a protein with 72% similarity to PccB from *S. coelicolor*. In partnership with AccA2, the putative YCC β-subunit encoded by *samR0483* has been proposed to be responsible for assembly of the unusual extender units incorporated into the stambomycins (Fig. 1)^2^. However, the biosynthesis of unusual PKS extender units by YCCs is without precedent. Moreover, the *S. ambofaciens* genome contains several genes encoding homologues of CCRCs, albeit not clustered with the stambomycin biosynthetic genes and predicted on the basis of sequence alignments to be incapable of directing the production of pentyl- or hexylmalonyl-CoAs (Supplementary Fig. 1). Thus, the biosynthetic mechanism for the unusual extender units incorporated into the stambomycins is unclear.

Here we report a series of experiments that elucidate the biosynthetic origin and mechanism of assembly of the unusual stambomycin PKS extender units. Incorporation of stable isotope-labelled precursors indicate that they originate from fatty acid metabolism. Retention of both deuterium atoms in the stambomycin analogue derived from feeding of [3-^2^H_2_]heptanoic acid to *S. ambofaciens* rules out the involvement of a CCRC in unusual extender unit biosynthesis. Direct evidence for the involvement of *samR0483* in this process is provided by expression of this gene in a *revT* mutant of *Streptomyces* sp. SN-593, in which the CCRC-dependent pathway for the biosynthesis of *n*-pentylmalonyl-CoA (and related) extender units incorporated into the reveromycins is abolished. X-ray crystal structures of SamR0483 alone and in complex with hexanoyl-CoA reveal the molecular basis for medium chain acyl-CoA recognition by this unusual YCC β-subunit, prompting us to rename it medium chain acyl-CoA carboxylase beta subunit (MccB). Taken together, these results establish the existence of a unique YCC-dependent pathway for unusual polyketide extender unit biosynthesis. Finally, the potential of this pathway to be harnessed for selective polyketide derivatization is demonstrated by feeding 6-azidohexanoic acid and 8-nonynoic acid to *S. ambofaciens*, resulting in production of the corresponding azide and alkyne-tagged stambomycin analogues.

## Results

### Metabolic origin of the C-26 side chains of the stambomycins

**Figure 2:**
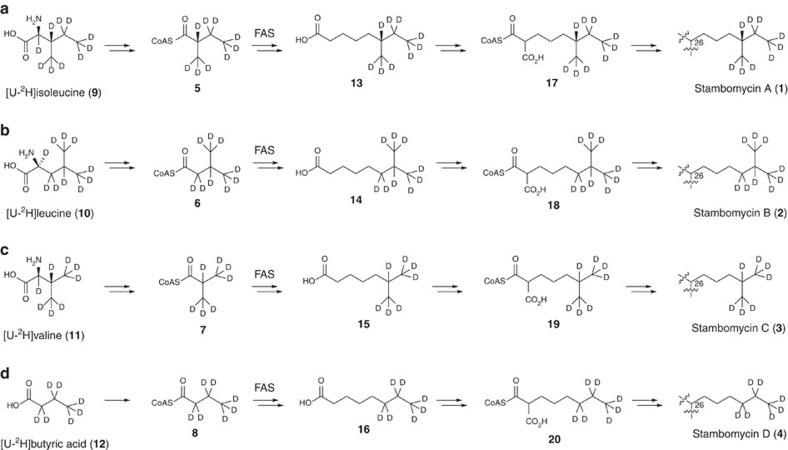
Incorporation of precursors of FAS starter units into the C-26 side chains of the stambomycins. (**a**) Incorporation of [U-^2^H]L-isoleucine **9** into the FAS starter unit 2-methylbutyryl-CoA **5**, the fatty acid 6-methyloctanoic acid **13**, the extender unit (4-methylhexyl)malonyl-CoA **17**, and stambomycin A **1**. (**b**) Incorporation of [U-^2^H]L-leucine **10** into the FAS starter unit 3-methylbutyryl-CoA **6**, the fatty acid 7-methyloctanoic acid **14**, the extender unit (5-methylhexyl)malonyl-CoA **18**, and stambomycin B **2**. (**c**) Incorporation of [U-^2^H]L-valine **11** into the FAS starter unit *iso*-butyryl-CoA **7**, the fatty acid 6-methylheptanoic acid **15**, the extender unit (4-methylpentyl)malonyl-CoA **19**, and stambomycin C **3**. (**d**) Incorporation of [U-^2^H]butyric acid **12** into the FAS starter unit *n*-butyryl-CoA **8**, the fatty acid *n*-octanoic acid **16**, the extender unit *n*-hexylmalonyl-CoA **20**, and stambomycin D **4**.

Stambomycins A-D **1**–**4** differ in the length and methylation pattern of their C-26 side chains^2^, which are of unknown biosynthetic origin. In *Streptomyces* species, the FabH component of the core metabolic fatty acid synthase (FAS) catalyses elongation of 2-methylbutyryl-, 3-methylbutyryl-, *iso*-butyryl-, and *n*-butyryl-CoA (**5-8**) starter units with malonyl-ACP^18^. Subsequent rounds of chain elongation are catalysed by FabF^19^. 2-Methylbutyryl-CoA (**5**), 3-methylbutyryl-CoA (**6**) and *iso*-butyryl-CoA (**7**) derive from transamination and oxidative decarboxylation of L-isoleucine, L-leucine and L-valine respectively (Fig. 2)^19^. We hypothesized that the C-26 side chains of the stambomycins are derived from **5**-**8**. To test this hypothesis, we fed universally deuterium labelled L-isoleucine (**9**), L-leucine (**10**), L-valine (**11**) and *n*-butyric acid (**12**) separately to cultures of *S. ambofaciens* W130 (a mutant of the ATCC23877 strain with an extra copy of *samR0484*, which encodes a pathway-specific transcriptional regulator of the stambomycin BGC, integrated into its chromosome under the control of the constitutive ermE* promoter). LC-ESI-TOF-MS analyses of mycelial extracts from these experiments showed that L-isoleucine and L-leucine are specifically incorporated into stambomyins A and B, whereas L-valine and *n*-butyric acid are specifically incorporated into stambomycins C and D, respectively (Fig. 2 and Supplementary Figs 2–5). These data are consistent with biosynthesis of the (4-methylhexyl), (5-methylhexyl), (4-methylpentyl) and *n*-hexylmalonyl-CoA extender units (**17**–**20**) incorporated into stambomycins A-D, respectively, from the corresponding fatty acids, that is 6-methyloctanoic acid (**13**), 7-methyloctanoic acid (**14**), 6-methylheptanoic acid (**15**) and *n*-octanoic acid (**16**) (Fig. 2). These fatty acids are probably converted to their CoA thioesters by SamR0482, a homologue of the medium chain fatty acyl-CoA ligase RevS, which was recently shown to participate in the biosynthesis of unusual extender units for the reveromycin PKS^11^.

### A mechanistic probe for unusual extender unit biosynthesis

**Figure 3:**
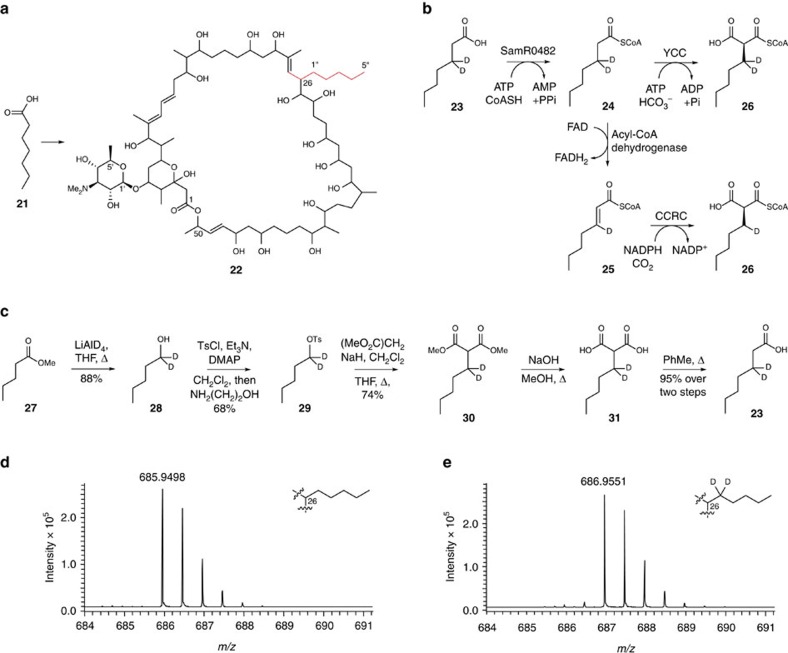
Discrimination between YCC and CCRC-dependent pathways using a mechanistic probe. (**a**) The novel stambomycin analogue **22** is produced when heptanoic acid **21** is fed to *S. ambofaciens* W130. The position of incorporation of heptanoic acid into the analogue is highlighted in red. (**b**) Concept underlying the development of a mechanistic probe to distinguish between CCRC- and YCC-dependent pathways for unusual alkylmalonyl-CoA PKS extender unit biosynthesis. Conversion of (3-^2^H_2_)heptanoic acid (**23**), via its CoA thioester **24**, to pentylmalonyl-CoA (**26**) by a YCC-dependent pathway would result in retention of both deuterium labels, whereas one of the deuterium labels would be lost in pentylmalonyl-CoA (**26**) formed by a CCRC-dependent pathway, because CoA thioester **24** must undergo desaturation to **25** in order to be reductively carboxylated. (**c**) Route employed for the synthesis of (3-^2^H_2_)heptanoic acid **23**. (**d**, **e**) Mass spectra of stambomycin analogue **22** from LC–MS analyses of mycelial extracts of *S. ambofaciens* W130 cultures supplemented with unlabeled heptanoic acid (**d**) and (3-^2^H_2_)heptanoic acid (**23**, **e**). The shift of one *m/z* unit for the doubly-charged parent ion in the right spectrum indicates that both deuterium labels are retained when (3-^2^H_2_)heptanoic acid (**23**) is incorporated into stambomycin analogue **22**.

Feeding of heptanoic acid (**21**) to *S. ambofaciens* W130 resulted in the production of a novel stambomycin derivative **22** with an *n*-pentyl side chain at C-26 (Fig. 3; Supplementary Figs 6–11), providing further support for the proposed fatty acid origin of the unusual extender units and highlighting the ability of the acyltransferase (AT) domain within module 12 of the stambomycin PKS to tolerate unnatural substrates. This inspired us to synthesize [3-^2^H_2_]heptanoic acid (**23**) as a mechanistic probe to determine whether the unusual extender units incorporated into the stambomycins are assembled by a CCRC or YCC-dependent pathway. Incorporation of [3-^2^H_2_]heptanoic acid (**23**) into the stambomycin analogue (**22**) via a CCRC-dependent pathway would be expected to proceed via initial SamR0482-catalysed conversion to the CoA thioester (**24**), followed by β-oxidation to yield the corresponding α, β-unsaturated thioester (**25**) and reductive carboxylation to yield *n*-pentylmalonyl-CoA (**26**) containing only a single deuterium label (Fig. 3). In contrast, the YCC-dependent pathway would proceed via direct carboxylation of [3-^2^H_2_]heptanoyl-CoA (**24**), resulting in *n*-pentylmalonyl-CoA (**26**) containing two deuterium labels (Fig. 3). Thus, both of the deuterium atoms would be expected to be retained in the stambomycin analogue if *n*-pentylmalonyl-CoA is biosynthesized via the YCC-dependent pathway, whereas one of the deuterium labels would be lost if this extender unit is assembled by the CCRC-dependent pathway.

[3-^2^H_2_]heptanoic acid **23** was synthesized in five steps from methyl-*n*-pentanoate **27** (Fig. 3; Supplementary Figs 12 and 13). Reduction of methyl-*n*-pentanoate **27** with LiAlD_4_ yielded [1-^2^H_2_]pentanol **28**, which was converted to its tosylate derivative **29** by reaction with TsCl, DMAP and Et_3_N. Deprotonation of dimethyl malonate with sodium hydride followed by alkylation with **29** gave **30**. Hydrolysis of **30** with sodium hydroxide in methanol afforded the corresponding diacid **31**, which was decarboxylated by refluxing in toluene to give yield the mechanistic probe **23**.

Comparison of the mass spectra for stambomycin analogue **22** from LC–ESI–TOF–MS analyses of mycelial extracts of *S. ambofaciens* W130 cultures that had been fed with labelled and unlabelled heptanoic acid showed that the [M+2H]^2+^ parent ions differ by one *m/z* unit (Fig. 3). Thus both atoms of deuterium are retained when [3-^2^H_2_]heptanoic acid **23** is incorporated into stambomycin analogue **22**, because the parent ion is doubly charged (Fig. 3; Supplementary Fig. 14). These results confirmed that the pentylmalonyl-CoA extender unit incorporated into stambomycin analogue **22** is biosynthesized via an YCC-dependent pathway.

**Figure 4:**
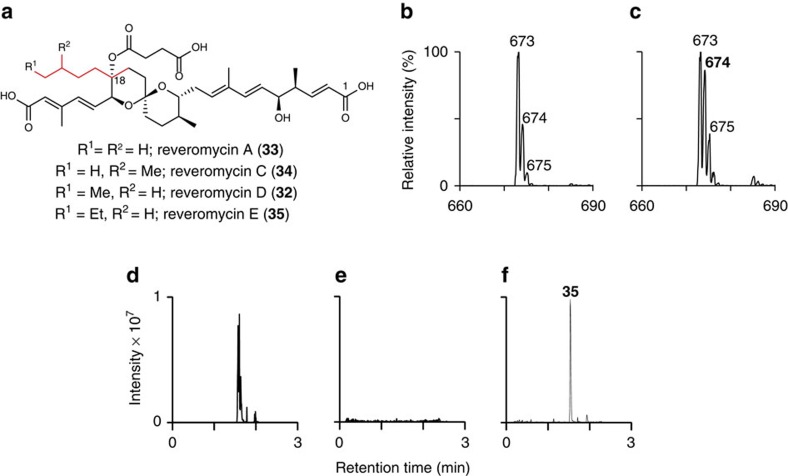
Reveromycins produced by *Streptomyces sp*. SN-593 wild type and *revT*::*samR0483* mutant. (**a**) Structures of the reveromycins produced by wild type *Streptomyces* sp. SN-593. The site of unusual alkylmalonyl-CoA extender unit incorporation is highlighted in-red. (**b**, **c**) Mass spectra of reveromycin D (**32**) from UHPLC-ESI-MS analyses of ethyl acetate extracts of *Streptomyces* sp. SN-593 *revR* mutant cultures grown in the absence (**b**) and presence (**c**) of (3-^2^H_2_)heptanoic acid (**23**). The increase in intensity of the *m/z*=674 peak in the right spectrum is consistent with incorporation of (3-^2^H_2_)heptanoic acid (**23**) into reveromycin D (**32**) with loss of one of the two deuterium labels. (**d**, **e**, **f**) Extracted ion chromatograms at *m/z*=687–688 corresponding to [M-H]^−^ for reveromycin E from UHPLC-ESI-MS analyses of ethyl acetate extracts of wild type *Streptomyces* sp. SN-593 (**d**), the *revT* mutant (**e**) and the *revT* mutant (**f**) in which *samR0483* has been expressed.

To further demonstrate the ability of our mechanistic probe to distinguish between CCRC and YCC-dependent pathways for unusual polyketide extender unit biosynthesis, we investigated the incorporation of [3-^2^H_2_]heptanoic acid **23** into revermomycin D **32**. *Streptomyces* sp. SN-593 produces reveromycins A, C, D and E (**33**, **34**, **32** and **35**), a family of structurally-related polyketides that differ in the nature of their C-18 side chains (Fig. 4)^20^. The structural differences between the reveromycins result from the utilization of various unusual extender units by module 4 of the reveromycin polyketide synthase^20^. The *revR*, *revS* and *revT* genes, which encode a FabH homologue, a medium chain fatty acyl-CoA ligase, and a CCRC, respectively, have recently been implicated in the biosynthesis of these extender units^11^. Reveromycin D **32** incorporates *n*-pentylmalonyl-CoA, but its abundance is low relative to reveromycin A **33**, which is derived from an *n*-butylmalonyl-CoA extender unit. Deletion of *revR* suppresses the production of reveromycin A **33** relative to the other congeners^11^. Thus, we fed [3-^2^H_2_]heptanoic acid **23** to a *revR* mutant of *Streptomyces* sp. SN-593. LC–MS analysis of an ethyl acetate extract of this culture showed that [3-^2^H_2_]heptanoic acid **23** was incorporated into reveromycin D **32** with retention of only a single deuterium label, consistent with the assembly of *n*-pentylmalonyl-CoA in *Streptomyces* sp. SN-593 via a CCRC-dependent pathway (Fig. 4)^11^. Moreover, high resolution ESI-MS analysis of partially purified reveromycins A, C and D confirmed that [3-^2^H_2_]heptanoic acid **23** is incorporated exclusively into reveromycin D **32** (Supplementary Fig. 15).

### Functional equivalence of SamR0483 and the CCRC RevT

In-frame deletion of *revT* in *Streptomyces* sp. SN-593 has been recently reported to abolish reveromycin production^11^. Expression of *samR0483* in the *revT* mutant restored the production of reveromycin E **35** (Fig. 4), demonstrating the ability of the YCC β-subunit encoded by this gene to biosynthesize *n*-hexylmalonyl-CoA. Only trace amounts of reveromycins A and D were observed in the *revT* mutant expressing *samR0483*. However, feeding of [3-^2^H_2_]heptanoic acid **23** to this strain resulted in production of a modest amount of reveromycin D **32** in which both of the deuterium labels were retained (Supplementary Fig. 16). This is consistent with assembly of the *n*-pentylmalonyl-CoA extender unit incorporated into reveromycin D **32** via an YCC-dependent pathway in the *revT*::*samR0483* mutant.

### X-ray crystal structure SamR0483

**Figure 5:**
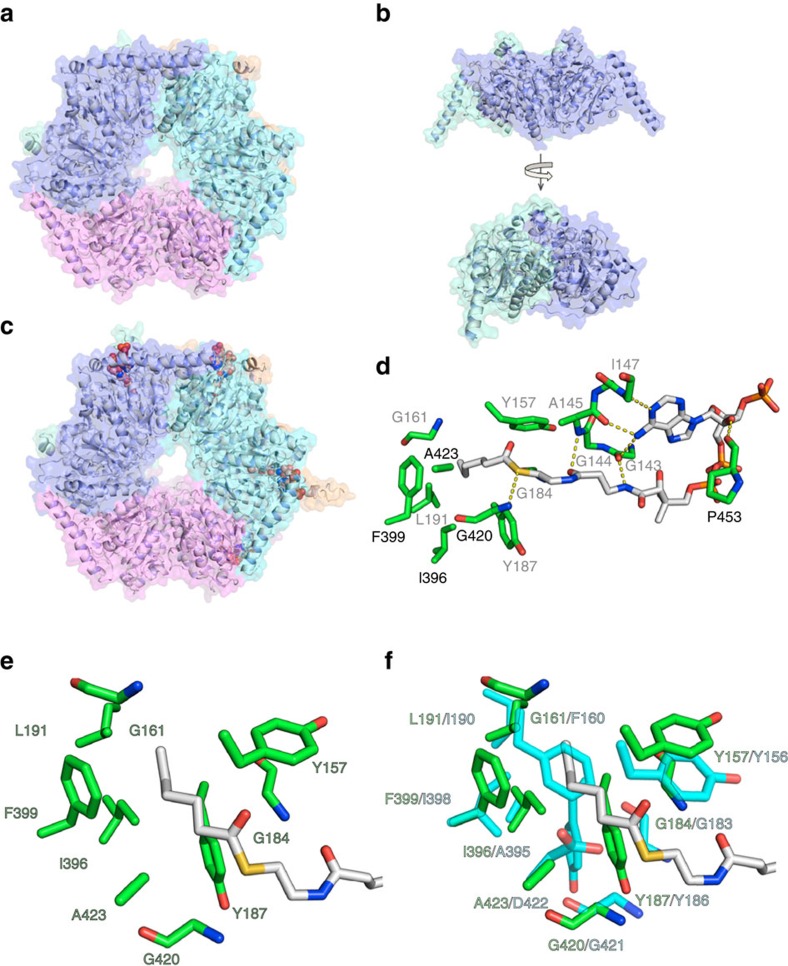
X-ray crystal structures of *apo*-MccB and *holo*-MccB. (**a**) The hexameric overall structure of *apo*-MccB. (**b**) The substrate binding site at the subunit interface of *apo*-MccB. (**c**) Structure of MccB with 4 molecules of hexanoyl-CoA bound, showing the overall architecture of the complex with hexanoyl-CoA in space filling representation. (**d**) Polar contacts between hexanoyl-CoA and MccB. Residues labelled in black correspond to monomer F and residues labelled in grey correspond to monomer B of the MccB homo-dimer. (**e**) Residues lining the hydrophobic pocket in MccB that bind the aliphatic chain of hexanoyl-CoA. (**f**) Overlay of the structures of MccB (green) and PccB (cyan), highlighting residues lining the binding pockets for the aliphatic chains of hexanoyl- and propionyl-CoA, their respective substrates.

To develop a better understanding of the molecular basis for the unique substrate specificity of the acyl-CoA carboxylase β-subunit encoded by *samR0483*, hereafter referred to as MccB, we solved its crystal structure to 2.45 Å. MccB was crystallized as a hexamer in the orthorhombic P2_1_2_1_2_1_ space group, and was solved via molecular replacement using the hexameric PccB D422A mutant structure (PDB ID: 3IBB) as a search model. Similar to other bacterial YCC β-subunits, MccB has 3–2 symmetry and is composed of three individual dimers that form a ring-like hexamer (Fig. 5). Each monomer can be divided into two domains harbouring two copies of the crotonase fold that pack together in an antiparallel manner to form a dimer (Fig. 5). The active site of MccB is located at the dimerization interface for two opposing monomers where residues 137–237 from the N-terminus of monomer B are packed against residues 392–492 from the C-terminus of the next proximal monomer F (Fig. 5). The individual monomers themselves superimpose well with a backbone Cα RMSD of 0.167 Å.

Overall, MccB has a high degree of structural similarity to the previously solved β-subunit of propionyl-CoA carboxylase PccB from *Streptomyces coelicolor*^21^, with a Cα backbone RMSD of 0.738 Å (Supplementary Fig. 17). Consequently, the vast majority of the known substrate and cofactor binding residues of YCC β-subunits are both structurally and sequentially conserved for MccB, PccB and AccD5 from *Mycobacterium tuberculosis* (Supplementary Fig. 18)^21^,^22^. The greatest structural deviations of MccB from previously solved structures occur near residues 33–46 and 452–477. Residues 33–46 of MccB form a long α-helix resulting in a larger cavity near the substrate binding zone. Residues 452–477 of MccB could not be modelled in the *apo* structure, highlighting this region's high degree of flexibility. In contrast, the region corresponding to residues 452–477 for PccB adopts a partially ordered helix–loop–helix motif. The high flexibility of MccB in the vicinity of the substrate binding pocket is consistent with its ability to bind larger acyl-CoA substrates than PccB.

### Molecular basis for hexanoyl-CoA recognition

Given the high degree of structural and sequence similarity between MccB and PccB, it was not immediately clear how the former is able to accommodate significantly longer acyl-CoA substrates than the latter. To address this question structures of MccB bound to either one or four molecules of hexanoyl-CoA were solved to 2.75 and 2.85 Å, respectively (Fig. 5; Supplementary Fig. 19). These co-crystal structures were obtained via incubation of MccB with 5 or 10 mM hexanoyl-CoA before crystallization. Interestingly, the electron density corresponding to residues 452–477 of monomer F was only present in the crystal form bound to four molecules of hexanoyl-CoA, suggesting that the helix-loop-helix lid region of MccB is stabilized at higher substrate concentrations.

The hexanoyl-CoA bound structures reveal that some of the substrate-binding features of MccB are similar to those employed by other YCC β-subunits. For example, the residues that interact with the adenine ring of the substrate are conserved between MccB and PccB. However, MccB and PccB recognize the phosphopantetheine (PPant) and acyl moieties of hexanoyl-CoA quite differently (see below).

The residues that interact with hexanoyl-CoA are localized in three regions at the MccB dimer interface. The first region is located at the entrance of the substrate binding pocket, which is characterized by a network of hydrogen bonds between the adenosine moiety of hexanoyl-CoA and the protein. The exocyclic amine and N-1 of adenine form hydrogen bond contacts with the peptide backbone of Ile147, Ala145 and Gly143 monomer B (Fig. 5). The adenosine moiety is further stabilized by a hydrogen bond from the backbone carbonyl group of Pro453 to the 2' hydroxyl group of ribose (Fig. 5). The second region is the PPant binding site, in which the backbone N-H groups of Ala145 and Gly184 in monomer B form hydrogen bonds to the carbonyl oxygen atom of the β-alanine component of PPant. In addition, the sulfur atom of the thioester is hydrogen bonded to the backbone N-H of Gly420 (Fig. 5). The third region is the binding pocket for the aliphatic portion of the substrate, which is formed primarily by the hydrophobic side chains of Ile396, Phe399 and Ala423 in Monomer F, and Leu191, Tyr157 and Tyr187 in monomer B (Fig. 5). Collectively, these three sets of interactions serve to anchor hexanoyl-CoA strongly into the active site of MccB.

The structures of MccB with hexanoyl-CoA bound were compared with the previously-reported structure of PccB bound to propionyl-CoA (Fig. 5)^21^. In the PccB co-crystal structure, propionyl-CoA binds close to the entrance of the active site, leaving the 3' phosphate of CoA more exposed to the solvent. In contrast, MccB sequesters its substrate deep into the active site via hydrophobic interactions between the side chains of the residues lining the substrate binding pocket and the aliphatic portion of hexanoyl-CoA. Further, the adenine ring of hexanoyl-CoA is tilted ∼90° with respect to that of the adenine-ring of propionyl-CoA in the PccB co-crystal structure. Residue 421 of PccB is Asp, whereas in MccB the corresponding residue is Ala. Mutation of Asp421 to Ala allows PccB to bind substrates with a longer aliphatic chain, such as butyryl-CoA^23^. This suggests that the negatively-charged Asp421 side chain of PccB diminishes the hydrophobic character of the binding pocket, thus biasing its substrate specificity towards acyl-CoAs with shorter aliphatic chains. In comparison, the MccB substrate binding pocket consists of more hydrophobic residues, with higher flexibility at the substrate entrance thus allowing it to accommodate longer aliphatic chains.

Further insight into the molecular basis for the unique substrate specificity of MccB was gleaned from sequence alignments and detailed structural comparisons with other YCC β-subunits (Fig. 5 and Supplementary Fig. 20). Structural alignment of MccB with PccB demonstrated that Gly161 in the former corresponds to a bulky Phe residue in the latter. Superimposition of the substrate bound MccB and PccB structures strongly suggested that replacing Gly161 with Phe in MccB would cause a steric clash with the aliphatic chain of hexanoyl-CoA (Fig. 5). Indeed, sequence alignments reveal that Phe is universally conserved in position 161 of all YCC β-subunits known to carboxylate acyl-CoAs with four carbons or less (Supplementary Fig. 20).

### Precursor-directed biosynthesis of stambomycin analogues

Inspired by the insights into the molecular basis for substrate specificity generated by our structural analysis of MccB, we sought to further probe the enzyme's substrate tolerance by investigating the precursor-directed biosynthesis of additional stambomycin analogues. In particular, we targeted the production of analogues bearing an alkyne or azide group at the terminus of the C-26 substituent, with the aim of carrying out click chemistry on these functional groups to produce biotinylated or fluorescently-labelled stambomycin derivatives for future mechanism of action studies.

**Figure 6:**
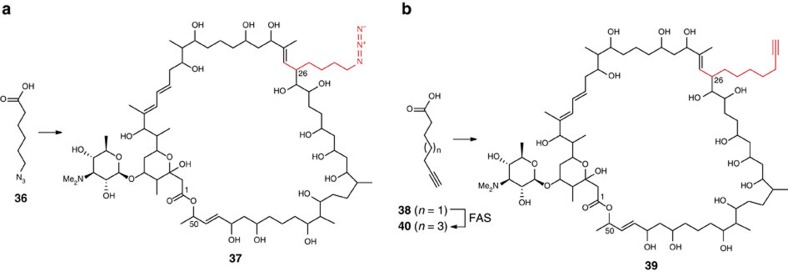
Bio-orthogonally-tagged stambomycin analogues produced by precursor directed biosynthesis. (**a**) Azide-tagged stambomycin analogue **37** is produced when 6-azidohexanoic acid **36** is fed to *S. ambofaciens* W130. (**b**) Acetylene-tagged stambomycin analogue **39** is produced when either 6-heptynoic acid **38** or 8-nonynoic acid **40** is fed to *S. ambofaciens* W130. 6-heptynoic acid **38** presumably undergoes conversion to its CoA thiosester, 2-carbon elongation by the primary metabolic FAS and hydrolysis to form 8-nonynoic acid **40** before incorporation into **39**.

Thus, we fed commercially available 6-azidohexanoic acid **36** to cultures of *S. ambofaciens* W130. UHPLC-ESI-TOF-MS analyses of mycelial extracts identified a novel metabolite with the molecular formula C_70_H_128_N_4_O_22_ (calculated for [C_70_H_129_N_4_NaO_22_]^2+^: 699.4414; found: 699.4419), corresponding to the stambomycin analogue **37** (Fig. 6; Supplementary Fig. 21). This analogue presumably arises from the conversion of 6-azidohexanoic acid **36** to its CoA thioester followed by MccB-mediated carboxylation to produce 4-azidobutylmalonyl-CoA, which is then utilized as an extender unit by module 12 of the stambomycin PKS. However, the production level of stambomycin analogue **37** is relatively modest compared with that of stambomycins A-D **1-4** (Supplementary Fig. 21), suggesting that azide-containing substrates are not well tolerated by SamR0482, MccB or stambomycin PKS module 12. Indeed, we were not able to obtain sufficient quantities of **37** for NMR spectroscopic analysis.

Surprisingly, feeding of commercially available 6-heptynoic acid **38** to *S. ambofaciens* W130 resulted in the production of a new metabolite with the molecular formula C_73_H_129_NO_22_ (calculated for [C_73_H_130_NNaO_22_]^2+^: 697.9486; found: 697.9505), corresponding to the stambomycin analogue **39** derived from 8-nonynoic acid (Fig. 6; Supplementary Fig. 22). This analogue presumably arises from 2-carbon elongation of 6-heptynoic acid **38** by the primary metabolic FAS of *S. ambofaciens*, yielding 8-nonynoic acid **40**, which is converted to its CoA thioester, carboxylated by MccB, and utilized as an extender unit by module 12 of the stambomycin PKS. To test this hypothesis, we synthesized 8-nonynoic acid **40** and fed it to *S. ambofaciens* W130. This resulted in the production of stambomycin analogue **39** at a similar level to that of stambomycins A and B combined (Fig. 6; Supplementary Fig. 23), allowing sufficient quantities of the analogue to be purified for ^1^H NMR spectroscopic analysis (Supplementary Fig. 24). The reaction of **39** with commercially available azide-PEG3-biotin resulted in conversion to the corresponding biotinylated compound (Supplementary Fig. 25), providing proof of principle for the production of bio-orthogonally-tagged stambomycin derivatives.

### YCCs assemble other unusual PKS extender units

**Figure 7:**
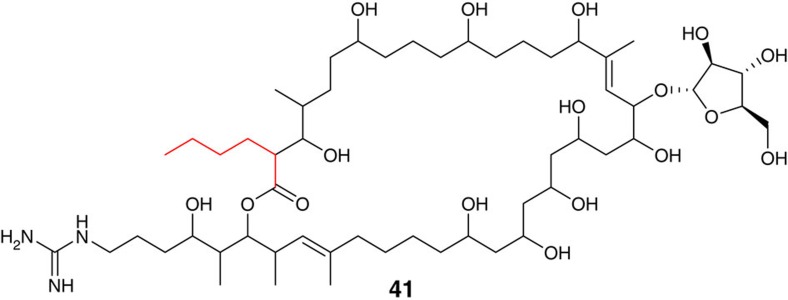
Structure of primycin A. The site of incorporation of the unusual butylmalonyl-CoA extender unit, which is proposed to be assembled by a YCC-dependent pathway, is highlighted in red.

BLAST searches identified two other polyketide biosynthetic gene clusters that contain genes encoding an YCC β-subunit with the same specificity-conferring residues as *S. ambofaciens* MccB (Supplementary Fig. 20). These clusters also contain genes encoding homologues of SamR0482. One of these genes clusters has recently been reported to direct the biosynthesis of the primycin complex of antibiotics^24^, which has been used in the clinic as a topical antibiotic and has recently been shown to be effective against multi-drug resistant Gram-positive pathogens, including methicillin- and mupirocin-resistant *Staphylococcus aureus*^25^. Butylmalonyl-CoA is incorporated into primycin A1 (**41**) (Fig. 7), and other members of the primycin complex appear to derive from similar 6, 7 and 8-carbon alkylmalonyl-CoA extender units^24^. It seems highly likely that these extender units are assembled by the sequential action of the SamR0482 and MccB homologues in the primycin producer.

The other gene cluster encoding MccB and SamR0482 homologues is cryptic (that is its metabolic product is unknown). It encodes only a single PKS module, and is widely conserved in *Amycolatopsis* species. The results presented herein suggest that it might be possible to produce an acetylene-tagged derivative of the metabolic product of this gene cluster which may facilitate purification and subsequent identification via a click-mediated biotinylation and avidin affinity purification strategy.

## Discussion

Several examples of unusual type I modular PKS extender units have been reported over the past half-decade. In all cases, these are assembled via a common final biosynthetic step, involving reductive carboxylation of an α, β-unsaturated CoA thioester by a CCRC homologue. The data reported here reveal an alternative mechanism for assembly of unusual type I modular PKS extender units. This employs a unique YCC β-subunit (MccB) that, in partnership with the primary metabolic YCC α-subunit, is able to directly carboxylate medium chain acyl-CoA thioesters. X-ray crystallographic analysis revealed the structural basis for substrate recognition by MccB, allowing a homologue likely involved in the biosynthesis of butylmalonyl-CoA and other unusual extender units incorporated into the primycin complex of clinically-used antibiotics to be identified.

These findings inspired the precursor-directed biosynthesis of novel azide- and alkyne-containing stambomycin analogues. Several biosynthetic engineering approaches have recently been utilized to introduce alkyne-terminated extender units containing 5–8 carbons into erythromycin and antimycin analogues^26^,^27^,^28^. Feeding of 8-nonynoic acid to *S. ambofaciens* resulted in the production of a stambomycin analogue containing a 9-carbon alkyne-terminated extender unit in comparable quantities to the natural products. Thus, MccB, the AT domain from module 12 of the stambomycin PKS and the medium chain acyl-CoA synthetase encoded by *samR0482* are useful additions to the synthetic biology toolkit for bio-orthogonal tagging of polyketides.

## Methods

### Bacterial strains and plasmids

The bacterial strains and plasmids used in the study are described in Supplementary Table 1 and the oligonucleotide primers used are described in Supplementary Table 2.

### Construction of *S. ambofaciens* W130

The LAL regulatory gene, *samR0484*, was PCR amplified from genomic DNA of *S. ambofaciens* ATCC23877 using the primers pOSV_samR0484_FW and pOSV_samR0484_RV and cloned into the *Cla*I/*Hin*dIII restriction sites of pOSV556t, placing *samR0484* under the control of the constitutive ermE* promoter. pOSV556t-*samR0484* was transferred into *S. ambofaciens* ATCC 23877 via conjugation from *E. coli* ET12567/pUZ8002 (ref. 2). One of the resulting hygromycin-resistant exconjugants was picked and designated as *S. ambofaciens* W130. Subsequent analysis^2^, showed that *S. ambofaciens* W130 overproduces the stambomycins.

### Incorporation of labelled precursors into the stambomycins

MP5 agar plates containing 5 mM [U-^2^H]L-valine **11**, [U-^2^H]butyric acid **12**, [U-^2^H]L-isoleucine **9**, or [U-^2^H]L-leucine **10** were overlaid with sterile permeable membranes (12–14,000 Da molecular weight cut off, size 20). A 4 μl aliquot of *S. ambofaciens* W130 spore stock was spread onto each plate and they were incubated at 30 °C for 4 days. The mycelia were scraped off the membranes and extracted with methanol. The extracts were analysed by UHPLC-ESI-TOF-MS on a Dionex 3,000 RS UHPLC attached to a Bruker MaXis mass spectrometer (Supplementary Figs 2–5). An Agilent Zorbax Eclipse plus column (C18, 100 × 2.1 mm, 1.8 μm) was used. Mobile phases consisted of A (water with 0.1% formic acid) and B (acetonitrile with 0.1% formic acid), the flow rate was 0.2 ml min^−1^ and absorbance at 240 nm was monitored. The elution profile was as follows: 0 min, 20% B; 5 min, 20% B; 20 min, 100% B. The mass spectrometer was operated in positive mode, with a scan range of 50–3,000 *m/z*, and calibrated with sodium formate (10 mM) through a loop injection of 20 μl at the beginning of each run. Source conditions were as follows. End plate offset: −500 V; capillary: −4,500 V; nebulizer gas (N_2_): 1.4 bar; dry gas (N_2_): 8 l/min; dry temperature: 180 °C. Ion tranfer conditions were as follows. Ion funnel 1 RF: 200 Vpp; ion funnel 2 RF: 200 vpp; hexapole RF: 200 Vpp; quadruple ion energy: 5 ev; quadrupole low mass: 55 m/z; collision energy: 5.0 ev; collision RF: ramped from 800 to 1,500 Vpp; transfer time: from 100 to 155 μs; pre-pulse storage time: 5 μs.

### Precursor directed biosynthesis of stambomycin analogue 22

Heptanoic acid **21** was fed to cultures of *S. ambofaciens* W130 as described above for incorporation of labelled precursors into the stambomycins. UHPLC-ESI-Q-TOF-MS analysis of the methanolic mycelial extracts, as described above, showed that a new metabolite with a molecular formula corresponding to **22** was produced (Supplementary Fig. 6).

The stambomycin analogue was purified from the mycelial extract by semi-preparative HPLC on an Agilent C18 column (100 × 21 mm, fitted with a C18 pre-column 10 × 21 mm) connected to an Agilent 1100 instrument. The mobile phases used were water (A) and methanol (B), both of which contained 0.1% formic acid. The total run time was 55 min with a flow rate of 5 ml/min and absorbance was monitored at 240 nm. The elution conditions employed during the run were as follows: 0 min, 60% A/40% B; 5 min, 60% A/40% B; 30 min, 100% B; 35 min, 100% B; 40 min, 60% A/40% B. Fractions containing the stambomycin analogue were identified by ESI-MS analysis, pooled and concentrated to dryness by rotary evaporation and lyophilisation. The residue was analysed by ^1^H, ^13^C-APT, COSY, TOCSY and HMBC NMR spectroscopy (Supplementary Table 3; Supplementary Figs 7–11).

### Synthesis of [3-^2^H_2_]heptanoic acid 23 and 8-nonynoic acid 40

*General experimental procedures*. All reagents (purchased from Sigma Aldrich, Alfa Aesar, Acros Organics or Jena Bioscience) and solvents were used as supplied, unless otherwise stated. THF and DCM were dried over calcium hydride, distilled and stored over 4 Å molecular sieves under argon. Dry triethylamine was obtained by distillation over calcium hydride. Dry dimethyl malonate was obtained by vacuum distillation at 37 mbar pressure at 90 °C under argon and stored over 4 Å molecular sieves under argon. Commercially available *p*-toluenesulphonyl chloride was recrystallized before use. To a solution of *p*-toluenesulphonyl chloride (25 g) in chloroform (100 ml) was added hexane (500 ml). The resulting mixture was stirred for 30 min, the white precipitate formed was removed *via* filtration and the solvent was removed under vacuum. Solvents were evaporated using a Buchi Rotavapor R-200 equipped with a Buchi Vacuubrand pump. Residual solvent was removed on a high vacuum line using an Edwards E2M5 vacuum pump. Flash chromatography was conducted on Fluka Silica Gel (40–63 μm, 60 Å). TLC was peformed on aluminium backed plates pre-coated with Merck silica gel (60 F_254_) and visualised by ultraviolet radiation and potassium permanganate. All glassware was dried by heating in a 140 °C oven for a minimum of 2 h.

Low resolution ESI mass spectra were recorded using a Bruker Esquire 2000 spectrometer and high resolution mass spectra were measured by the University of Warwick Mass Spectrometry service on a Bruker MaXis ESI-Q-TOF mass spectrometer. NMR spectra were obtained using a Bruker DPX400 (^1^H 400 MHz and ^13^C 100 MHz) equipped with a proton-carbon dual probe. High field spectra were measured by the University of Warwick NMR Service using Bruker AV III-600 (^1^H 600 MHz and ^13^C 150 MHz) or AV II-700 (^1^H 700 MHz and ^13^C 175 MHz) instruments. Chemical shifts are quoted in ppm with respect to the residual solvent peak. Deuterated solvents (Sigma Aldrich) were used as supplied.

*[1-*^*2*^*H*_*2*_*]n-pentanol **28***. To a stirred suspension of lithium aluminium deuteride (1.96 g, 51.7 mmol, 3 equiv) in 25 ml of dry THF under argon was added methyl valerate (2.25 ml, 17.2 mmol, 1 equiv) in 5 ml of dry THF drop wise. The resulting mixture was heated to 70 °C and the reaction was monitored by TLC (25% ethyl acetate in 40–60 °C pet. ether). Upon completion, the reaction was cooled to 0 °C and quenched with 2 M HCl, resulting in a white precipitate which was removed by vacuum filtration. The filtrate was concentrated *in vacuo* and diluted with 250 ml of water. The resulting solution was extracted with 3 × 100 ml ethyl acetate. The organics were combined, washed with brine and dried over magnesium sulfate. The solvent was removed *in vacuo* to yield the crude product, which was purified by flash column chromatography (silica, 25% diethyl ether in pentane) to yield the product as a colourless oil (1.37 g, 88%). ^1^H NMR (400 MHz, CDCl_3_): *δ* 1.54 (*t*, 2H, *J*=6.5 Hz), 1.32–1.28 (*m*, 4H,), 0.86 (*t*, 3H, *J*=6.5 Hz); ^13^C NMR (100 MHz, CDCl_3_): *δ* 62.3 (sep, *J*=19.7 Hz), 32.4, 28.0, 22.5, 14.0.

*[1-*^*2*^*H*_*2*_*]n-pentane 4-methylbenzenesulfonate* ***29***. To a stirred solution of [1-^2^H_2_]*n*-pentanol **28** (1.17 g, 12.9 mmol, 1 equiv) in 23 ml of dry DCM, under argon, was added 4-dimethylamino-pyridine (473 mg, 3.88 mmol, 0.3 equiv) and triethylamine (3.27 g, 32.3 mmol, 2.5 equiv) followed by *p*-toluene sulfonyl chloride (2.71 g, 14.4 mmol, 1.1 equiv). The reaction was stirred at room temperature under argon for 17 h, after which TLC indicated that it had gone to completion. Ethanolamine (1.58 g, 26.6 mmol, 2 equiv) was added and the resulting mixture was stirred for a further 2 h. 125 ml of water was added, the layers were separated and the aqueous layer was extracted with 4 × 50 ml of DCM. The organics were combined, washed with brine and dried over magnesium sulfate. The solvent was removed *in vacuo* and the residue was purified via flash column chromatography (silica, 10% ethyl acetate in 40–60 °C pet ether) to give the product as a colourless oil (2.13 g, 68%). ^1^H NMR (400 MHz, CDCl_3_): *δ* 7.79 (*d*, 2H, *J*=8.0 Hz), 7.34 (*d*, 2H, *J*=8.0 Hz), 2.44 (*s*, 3H), 1.62 (*t*, 2H, *J*=7.0 Hz), 1.28–1.23 (*m*, 4H), 0.84 (t, 3H, *J*=7.0 Hz); ^13^C NMR (100 MHz, CDCl_3_): *δ* 144.7, 133.3, 129.8, 127.9, 70.2 (sep, *J*=22.7 Hz), 28.3, 27.4, 22.1, 21.7, 13.8. HRMS (*m/z*) [M+H]^+^ calculated for C_12_H_15_D_2_NaO_3_S, 267.0994; found, 267.0990.

*dimethyl ([1-*^*2*^*H*_*2*_*]n-pentyl)malonate ***30**. To a stirred suspension of sodium hydride (471 mg, 19.7 mmol, 14.8 equiv) in 10 ml of dry DCM, under argon, was added distilled dimethyl malonate (2.70 g, 20.5 mmol, 5 equiv) in 15 ml dry of THF drop wise. The resulting mixture was stirred at 60 °C for 1 h and [1-^2^H_2_]*n*-pentyl-4-methylbenzenesulfonate **29** (1.00 g, 4.09 mmol, 1 equiv) in 10 ml of dry THF was added. Stirring was continued at 60 °C under argon for a further 36 h, after which TLC indicated that the reaction had gone to completion. 125 ml of water was added cautiously and the aqueous layer was extracted with 4 × 100 ml of ethyl acetate. The organics were combined, washed with brine and dried over magnesium sulfate. The solvent was removed *in vacuo* and the residue was purified by flash column chromatography (silica, 25% ethyl acetate in 40–60 °C pet ether), yielding the product as a colourless oil (616 mg, 74%). ^1^H NMR (400 MHz, CDCl_3_): *δ* 3.69 (*s*, 6H), 3.30 (*s*, 1H), 1.29–1.22 (6H, *m*), 0.84 (*t*, 3H, *J*=7.5 Hz); ^13^C NMR (100 MHz, CDCl_3_): *δ* 170.0, 52.4, 51.6, 31.3, 28.2 (sep, *J*=20.0 Hz), 26.8, 22.3, 13.9; HRMS (*m/z*): [M+Na]^+^ calculated for C_10_H_16_D_2_NaO_4_, 227.1223; found, 227.1221.

*[3-*^*2*^*H*_*2*_*]n-heptanoic acid **23***. To a stirred solution of dimethyl ([1-^2^H_2_]*n*-pentyl)malonate **30** (500 mg, 2.45 mmol, 1 equiv) in 10 ml of methanol, was added 5 M sodium hydroxide (2.9 ml, 14.7 mmol, 6 equiv) drop wise. The resulting mixture was stirred under reflux for 18 h, after which time TLC indicated that no starting material remained. The mixture was cooled to room temperature, acidified with 2 M HCl and the methanol was removed *in vacuo*. 100 ml of 20% brine was added to the residue and the resulting solution was extracted with 3 × 75 ml of ethyl acetate. The organics were combined, dried over magnesium sulfate and the solvent was removed *in vacuo* to yield diacid **31** as a crystalline solid. 15 ml of toluene was added and the resulting mixture was heated under reflux, at 125 °C for 96 h. The mixture was cooled to room temperature and the toluene was removed *in vacuo* to give the product (306 mg, 95%). ^1^H NMR (600 MHz, CDCl_3_): *δ* 2.25 (s, 2H), 1.29–1.24 (*m*, 6H) 0.80 (*t*, 3H, *J*=7.0 Hz); ^13^C NMR (150 MHz, CDCl_3_): *δ* 180.5, 34.1, 31.5, 28.7, 24.1 (sep, *J*=19.7 Hz), 22.6, 14.2; HRMS (*m/z*): [M-H]^−^calculated for C_7_H_12_D_2_O_2_, 131.1047; found, 131.1046. The ^1^H and ^13^C NMR spectra for labelled and unlabelled *n*-heptanoic acid are compared in Supplementary Figs 12 and 13.

*8-nonyn-1-ol*. Sodium hydride (8.56 g, 357 mmol) was added to ethylenediamine (200 ml) under argon and the resulting mixture was heated to 60 °C for 60 min. 3-Nonyl-1-ol (10.0 g, 71.4 mmol) was added and the resulting deep blue solution was stirred for 4 h, then cooled to 0 °C and carefully quenched by addition of 2 M HCl until the blue colour disappeared. The mixture was extracted with EtOAc, and the combined organics were dried (MgSO_4_) and concentrated under vacuum. The crude product was purified on silica gel (25% EtOAc in hexanes) to give the product (6.17 g, 61% yield) as a colourless oil.

^1^H NMR (700 MHz, CDCl_3_): *δ* 3.64 (*t*, 2H, *J*=6.5 Hz), 2.18 (td, 2H, *J*=7.0, 3.0 Hz), 1.96 (*t*, 1H, *J*=3.0 Hz), 1.57 (*m*, 2H), 1.53 (*m*, 2H), 1.44–1.31 (*m*, 6H). ^13^C NMR (175 MHz, CDCl_3_): *δ* 84.7, 68.1, 63.0, 32.7, 28.9, 28.7, 28.4, 25.6, 18.4; HRMS (*m/z*): [M+Na]^+^ calculated for C_9_H_15_ONa, 163.1099; found, 163.1096.

*8-nonynal*. To a solution of oxalyl chloride (5.00 ml, 57.2 mmol) in dry DCM (110 ml), cooled to −78 °C under argon, was added DMSO (7.50 ml, 106 mmol) in dry DCM (25 ml) drop wise. The reaction mixture was stirred for 5 min, then 8-nonyn-1-ol (6.17 g, 44.0 mmol) in dry DCM (50 ml) was added and stirring was continued for a further 15 min. Dry triethylamine (26.8 ml, 220 mmol) was added and the reaction mixture was allowed to warm to room temperature over 30 min. Water was added and the mixture was extracted with DCM. The combined organics were washed with 2 M HCl and saturated NaHCO_3_, dried (MgSO_4_), and concentrated under vacuum. The crude product was purified on silica gel (10% EtOAc in hexanes) to give the product (3.13 g, 51% yield) as a colourless oil.

^1^H NMR (300 MHz, CDCl_3_): *δ* 9.55 (s, 1H,), 2.42 (td, 2H, *J*=8.0, 2.0 Hz), 2.17 (td, 2H, *J*=7.0, 3.0 Hz), 1.92 (*t*, 1H, J=3.0 Hz), 1.62 (tt, 2H, *J*=8.0, 8.0 Hz), 1.57–1.28 (m, 6H); ^13^C NMR (175 MHz, CDCl_3_): *δ* 202.8, 84.5, 68.3, 43.8, 28.6, 28.4, 28.2, 21.9, 18.3; HRMS (*m/z*): [M+Na]^+^ calculated for C_9_H_13_ONa, 161.0937; found, 161.0939.

*8-nonynoic acid ****40***. To a solution of 8-nonynal (3.13 g, 22.7 mmol) in DMF (30 ml) was added Oxone (15.3 g, 24.9 mmol). The resulting suspension was stirred for 15 min, then poured into water, acidified with 2 M HCl and extracted with EtOAc. The combined organics were dried (MgSO_4_) and concentrated under vacuum to give the product (3.32 g, 95% yield) as a colourless oil.

^1^H-NMR (700 MHz, CDCl_3_): *δ* 2.29 (*t*, 2H, *J*=7.5 Hz), 2.12 (td, 2H, *J*=7.0, 2.5 Hz), 1.87 (*t*, 1H, J=2.5 Hz), 1.59 (tt, 2H, J=7.0, 7.0 Hz), 1.47 (tt, 2H, *J*=7.5, 7.5 Hz), 1.40–1.27 (m, 4H). ^13^C-NMR (CDCl_3_, 175 MHz): *δ* 179.6, 84.5, 68.3, 33.9, 28.5, 28.3, 28.2, 24.5, 18.3; HRMS (*m/z*): [M+Na]^+^ calculated for C_9_H_13_O_2_Na, 177.0886; found, 177.0884.

### Incorporation of [3-^2^H_2_]*n*-heptanoic acid 23 into 22

[3-^2^H_2_]*n*-heptanoic acid **23** was fed to cultures of *S. ambofaciens* W130 and the mycelial extract was prepared as described above. UHPLC-ESI-Q-TOF-MS analysis of the extract, as described above, showed that both of the deuterium atoms had been retained in the resulting **22** (Supplementary Fig. 14).

### Incorporation of [3-^2^H_2_]*n*-heptanoic acid 23 into 32

The Δ*revR* and Δ*revT::samR0483* mutants (Supplementary Table 1) were grown at 28 °C on MS agar (2% (w/v) soy flour, 2 % (w/v) D-mannitol, 2% (w/v) agar). Spores were used to inoculate a 500 ml cylindrical flask containing 70 ml of SY medium (0.1% (w/v) yeast extract (Difco), 0.1% (w/v) NZ-amine (Wako) and 1% (w/v) starch; pH 7.0) and the resulting pre-culture was grown for 2 days at 28 °C at 150 rpm. 1 ml of the pre-culture was used to inoculate a 500 ml cylindrical flask containing 70 ml of production medium (RM-PM; 2% (w/v) potato dextrose (Difco), 1% (w/v) malt extract (Difco), 1% (w/v) dried yeast (Asahi beer), 5% (w/v) tomato juice (Table land, Maruzen Food), 0.1%(w/v) K_2_HPO_4_, 0.1% (w/v) NaCl, 0.03% (w/v) MgSO_4_·7H_2_O, 0.01% (w/v) NaNO_3_, 0.005% (w/v) ZnSO_4_·7H_2_O and 0.005% (w/v) CuSO_4_·5H_2_O; pH 6.5 before autoclaving). After 2 days growth at 28 °C and 150 r.p.m., 0.3 mM of [3-^2^H_2_]*n*-heptanoic acid **23** was added and the culture was grown for three further days. 4 ml of culture broth was extracted with an equal volume of acetone and residual acetone was removed by evaporation. The pH was adjusted to 4 by adding acetic acid and the culture broth was extracted twice with an equal volume of ethyl acetate. The organic layer was concentrated *in vacuo* and dissolved in 1.2 ml of methanol. The resulting sample from the Δ*revR* mutant culture was analysed by UPLC-ESI-MS on an Applied Biosystems API 3200 connected to a Waters ACQUITY UPLC and a Waters ACQUITY UPLC BEH C18 column (1.7 μm, 2.1 mm internal diameter × 50 mm). The conditions were as follows: flow rate 0.7 ml min^−1^; solvent A, water containing 0.05% formic acid; and solvent B, acetonitrile. After injection of 1 μl of the sample into a column that was equilibrated with 30% solvent B, the column was developed with a linear gradient from 30% to 100% solvent B in 1.9 min and kept at 100% solvent B for 0.96 min. Mass spectra were recorded in negative ion mode.

To separate reveromycin D **32** from other reveromycin derivatives, the ethyl acetate extracts from the Δ*revR* and Δ*revT::samR0483* mutants were applied to a PEGASIL ODS column (5 μm, 4.6 × 250 mm). The HPLC conditions were as follows: flow rate, 1 ml min^−1^; solvent A, water containing 0.05% formic acid; and solvent B, MeOH, isocratic system of 78% MeOH. The fraction containing reveromycin D **32** from the Δ*revT::samR0483* mutant cultures with and without supplementation of (3-^2^H_2_)-heptanoic acid **23** was concentrated and analysed by UPLC-ESI-MS on an Applied Biosystems API 3200 connected to a Waters ACQUITY UPLC and a Waters ACQUITY UPLC BEH C18 column (1.7 μm, 2.1 × 50 mm). The flow rate was 0.2 ml min^−1^ and the column was eluted with isocratic 50% water containing 0.05% formic acid and 50% acetonitrile. Mass spectra were recorded in negative ion mode (Supplementary Fig. 16).

Partially purified reveromycin D **32** from the Δ*revR* mutant cultures with and without supplementation of [3-^2^H_2_]-heptanoic acid **23** was further analysed by negative ion ESI-TOF-MS on a Waters Synapt G2 mass spectrometer (Supplementary Fig. 15).

### Expression of *samR0483* in *Streptomyces sp*. SN-593 ΔrevT

The *samR0483* gene was amplified by PCR from genomic DNA of *S. ambofaciens* ATCC 23877 using the primers pOSV_samR0483_FW and pOSV_samR0483_RV and cloned into the *Cla*I/*Hin*dIII restriction sites of pOSV556t. The resulting plasmid, pOSV556t-*samR0483*, was used as the template for PCR amplification of *samR0483* using the primers 0483_BamHI and 0483_HindIII. The amplimer was cloned into the *Bam*HI and *Hin*dIII sites of pTYM19-P_*aph*_ yielding pTYM19*-*P_*aph*_*-samR0483*, which was used to transform *E. coli* GM2929 *hsdS::*Tn10 (pUB307::Tn7). pTYM19*-*P_*aph*_*-samR0483* was transferred by intergenic conjugation from *E. coli* GM2929 *hsdS::*Tn10 (pUB307::Tn7) to *Streptomyces* sp. SN-593 (ref. 29).

### Cloning of *samR0483* into *E. coli* expression vectors

The MccB-encoding gene, *samR0483*, was amplified by PCR from genomic DNA of *S. ambofaciens* ATCC 23877 using the primers TOPO_483_FW and TOPO_483_RV, and cloned into pET151 (Invitrogen) following the manufacturer's instructions to give pET151-*samR0483*. It was then amplified from pET151-*samR0483* using the primers pET28a-samR0483_FW and pET28a-samR0483_RV and the resulting amplimer was cloned into the *Nhe*I and *Not*I sites of pET28a (Novagen) to produce pET28a-*samR0483* encoding an N-terminal hexahistidine MccB fusion protein.

### Overproduction and purification of MccB

*E. coli* BL21(DE3) cells containing pET28a-*samR0483* were grown in 4 × 1L of LB medium supplemented with kanamycin (50 μg ml^−1^) to A_600_=0.6–0.8 at 37 °C and 180 r.p.m. Cultures were induced by the addition of 0.7 mM IPTG and incubated for 16 h at 19 °C and 180 r.p.m. Cells were harvested by centrifugation at 5,500 RCF for 10 min, re-suspended in lysis buffer (50 mM Tris–HCl pH 8.0, 300 mM NaCl, 20 mM imidazole and 10% glycerol), sonicated and the cell debris was removed by centrifugation (21,000 RCF for 70 min). The clarified cell lysate was batch bound to 5 ml of Ni-IMAC resin (Bio-Rad Laboratories) for 1 h at 4 °C. The resulting slurry was transferred to a gravity-flow column and washed twice with 50 ml of lysis buffer. His_6_-MccB was eluted with 5 ml fractions containing 40, 100, 150, 250 and 450 mM imidazole in lysis buffer. Protein purity was assessed by SDS–polyacrylamide gel electrophoresis (SDS–PAGE). Fractions containing the highest purity (≥90%) were collected and dialyzed overnight at 4 °C against 50 mM Tris–HCl pH 8.0, 50 mM NaCl, 10% glycerol and 1 mM DTT. Dialyzed protein was concentrated to 5–10 mg ml^−1^ using a 30,000 Da molecular weight cutoff centrifugal filter (Millipore) and injected onto a Superdex 200 column (GE Healthcare) pre-equilibrated with 50 mM Tris pH 8.0 and 1 mM DTT. Fractions were collected, analysed by SDS–PAGE for purity, concentrated to 9 mg ml^−1^ and flash frozen for storage at −80 °C.

For co-crystal studies, native His_6_-MccB was treated with TEV protease (10 U per mg of MCC) post nickel affinity chromatography and dialyzed into 50 mM Tris·HCl pH 8.0, 50 mM NaCl, 10% glycerol, 1 mM DTT, and 1 mM EDTA at 18 °C for 18 h. Complete digestion was verified using MALDI-TOF mass spectrometry. The cleaved protein was dialyzed into crystallization buffer (50 mM Tris pH 8.0 and 1 mM DTT) and subjected to anion exchange chromatography (High Trap Q, GE Healthcare) to remove excess TEV protease. Cleaved MccB was eluted using a linear gradient of 0–1 M NaCl and was dialyzed into 50 mM Tris pH 8.0 and 1 mM DTT for co-crystallization screens.

Selenomethionine (SeMet)*-*derivatized His_6_-MccB was produced in *E. coli* BL21(DE3) cells harbouring pET28a-*samR0483*. Cells were grown in 4 × 1 l of LB medium supplemented with kanamycin (50 μg ml^−1^) at 37 °C and 180 rpm until A_600_ reached 0.6. Before induction, the cells were harvested by centrifugation at 5,500 RCF for 10 min, re-suspended and washed three times with 100 ml of M9 medium then transferred to 4 × 1 l flasks containing M9 medium with the following additives: kanamycin (50 μg ml^−1^); lysine, phenylalanine, threonine (100 mg L^−1^); isoleucine, leucine, valine (50 mg L^−1^); and L-SeMet (80 mg L^−1^)(Sigma). The temperature was then lowered to 19 °C and protein expression was induced upon the addition of 0.7 mM IPTG. The SeMet substituted protein was purified following the same procedure as native His6-MccB. Selenomethionine incorporation was confirmed using MALDI-TOF mass spectrometry.

### Crystallization of *apo*- and hexanoyl-CoA-bound forms of MccB

Apo*-MccB crystallization*. Frozen SeMET aliquots of His_6_-MccB were thawed on ice and incubated with 5 mM biotin and 5 mM heptanoic acid (50 mM stocks in 0.1 M Hepes pH 7.2 and 100% DMSO, respectively) (Sigma-Aldrich) at 4 °C for 1 h before crystallization screens. Small crystals of *apo*-MccB formed overnight in 30% methyl propanediol (MPD) (Qiagen), 0.2 M magnesium acetate and 0.1 M MES pH 6.5. Diffraction-quality crystals were obtained by mixing 2 μl of protein solution with 2 μl of well solution (5–11% MPD, 0.2 M magnesium acetate and 0.1 M MES pH 6.5–6.8) using the hanging drop vapour diffusion method. *Apo*-MccB crystals were further optimized via four consecutive rounds of seeding using a Seed Bead (Hampton Research). The crystals were harvested, cryo-protected with 25% ethylene glycol and flash frozen in liquid nitrogen for subsequent X-ray diffraction data collection.

*MccB Hexanoyl-CoA co-crystallization*. Before crystallization, cleaved native MccB at 7 mg ml^−1^ was incubated with 5 mM biotin and either 5 mM or 10 mM hexanoyl-CoA (50 mM stock in 0.1 M Hepes pH 7.2) (Sigma-Aldrich). Crystals of the MccB-hexanoyl-CoA complex were obtained by mixing 2 μl of protein solution with 2 μl of mother liquor (26–32% PEG 5,000 MME, 0.2 M ammonium sulfate and 0.1 MES pH 6.5–7.0) using the hanging drop vapour diffusion method. MccB co-crystals grew at 25 °C over a period of 5 days. MccB co-crystals were cryo-protected in well solution plus 25% glycerol and flash frozen in liquid nitrogen for subsequent X-ray diffraction data collection.

### Collection and analysis of X-ray diffraction data

Data were collected at the Advanced Light Source (ALS), Lawrence Berkeley National Laboratory, using beamlines 8.2.1 or 8.2.2. Data sets corresponding to the *apo* and mono-hexanoyl-CoA co-crystal forms of MccB were indexed, integrated, and scaled using Mosflm or HKL2000, respectively^30^,^31^. Two separate data sets from a single crystal were collected for the tetra-hexanoyl-CoA MccB co-crystal form with 1 and 2 s exposure times. The individual data sets were processed using Mosflim and scaled together using Aimless^32^. Phasing of *apo*-MccB was accomplished via molecular replacement (Phaser^33^) using the hexameric *apo*-PccB D422A structure (PDB ID: 3IBB) as a search model. An initial model was built using Autobuild, and this model was further improved through iterative rounds of model building (COOT (ref. 34)) and refinement (PHENIX.REFINE^35^). For the co-crystal structures, electron density corresponding to hexanoyl-CoA was identified using PHENIX LigandFit and ligand restraints were generated using PHENIX eLBOW. Both the *apo* and co-crystal structures were validated using PHENIX.VALIDATE and PDB_REDO^36^,^37^. Resolution cutoffs were made based on a combination I/sigma, CC1/2, and per cent completeness values^37^. Crystallographic statistics for the three MccB crystal forms are listed in Supplementary Table 4 and the 2F0-FC SA omit maps for these structures and for the bound hexanoyl-CoA are shown in Supplementary Figs 19 and 26–28.

### Precursor directed biosynthesis of 37 and 39

6-azidohexanoic acid **36**, 6-heptynoic acid **38** and 8-nonynoic acid **40** were fed to cultures of *S. ambofaciens* W130 as described for precursor directed biosynthesis of stambomycin analogue **22**. UHPLC-ESI-QTOF-MS analysis of the methanolic mycelial extracts, as described for precursor directed biosynthesis of stambomycin analogue **22**, showed that new metabolites with molecular formulae corresponding to stambomycin analogues **37** (Supplementary Fig. 21) and **39** (Supplementary Figs 22 and 23) were produced.

Stambomycin analogue **39** was purified from the mycelial extract as described for stambomycin analogue **22** and analysed by ^1^H NMR spectroscopy (Supplementary Fig. 24).

### Reaction of 39 with azide-PEG3-biotin conjugate

To stambomycin analogue **39** (4.5 mg, 3.28 μmoles) suspended in degassed methanol (600 μl) and butanol (400 μl) under argon was added copper sulfate (0.1 mg, 0.65 μmoles, 0.2 equiv) and sodium ascorbate (0.65 mg, 3.28 μmoles, 1 equiv) in degassed water (1 ml), followed by azide-PEG3-conjugate (1.46 mg, 3.28 μmoles, 1 equiv) in degassed butanol (200 μl). Following 64 h stirring at 21 °C, the solvent was removed *in vacuo* and the biotinylated stambomycin derivative was purified by semi-preparative HPLC on an Agilent C18 column (100 × 21 mm, fitted with a C18 pre-column 10 × 21 mm). Elution conditions were as follows: flow rate, 5 ml min^−1^; solvent A, water containing 0.1% formic acid; solvent B, methanol containing 0.1% formic acid; gradient initiated after 5 min from 40% B to 100% B over 25 min. The biotinylated stambomycin derivative eluted between 18.6 and 19.2 min. 2 mg (34%) of the biotinylated stambomycin derivative was recovered and analysed by HRMS (*m/z* calculated for C_91_H_162_N_7_NaO_27_S^+^: 621.0339 [M+H+2Na]^3+^; Found: 621.0337) and ^1^H NMR spectroscopy (Supplementary Fig. 25).

### Data availability

X-ray crystallographic data have been deposited in the PDB under the accession codes 5INI, 5INF and 5ING. All other data are included in the manuscript (as figure source data or Supplementary Information) or are available from the authors on request.

## Additional information

**How to cite this article:** Ray, L. *et al*. A crotonyl-CoA reductase-carboxylase independent pathway for assembly of unusual alkylmalonyl-CoA polyketide synthase extender units. *Nat. Commun.* **7**, 13609 doi: 10.1038/ncomms13609 (2016).

**Publisher's note:** Springer Nature remains neutral with regard to jurisdictional claims in published maps and institutional affiliations.

## Supplementary Material

Supplementary Information Supplementary Figures 1-28, Supplementary Tables 1-4 and Supplementary References.

## Figures and Tables

**Figure 1:**
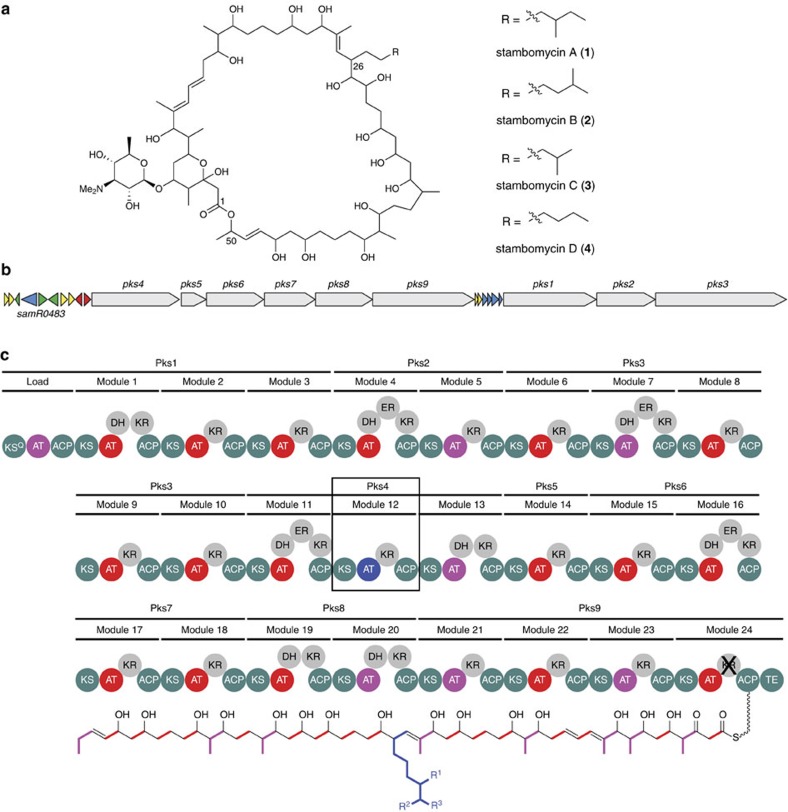
The stambomycin structures, their biosynthetic gene cluster in *S. ambofaciens* ATCC23877 and the modular PKS it encodes. (**a**) Structures of stambomycin A-D. The major constituents of the complex, stambomycins A-D (**1-4**), differ in the nature of their C-26 side chains. (**b**) Organization of the stambomycin biosynthetic gene cluster, highlighting the location of the *samR0483* gene encoding an acyl-CoA carboxylase β-subunit proposed to be involved in unusual extender unit biosynthesis. (**c**) Module and domain organization of the stambomycin PKS and structure of the polyketide chain it assembles. Carbon atoms derived from malonyl-CoA extender units are highlighted in red, while those derived from a methylmalonyl-CoA starter unit and methylmalonyl-CoA extender units are highlighted in purple. The carbon atoms derived from the unusual extender units utilized by PKS module 12 (boxed) are highlighted in blue (R^1^, R^2^ and R^3^=Me or H, depending on which of the unusual extender units is incorporated in a particular round of chain assembly). The acyltransferase (AT) domains responsible for loading of each of these building blocks onto the adjacent acyl carrier protein (ACP) domains are colour-coded accordingly.
