# Supplementary material for: A crotonyl-CoA reductase-carboxylase independent pathway for assembly of unusual alkylmalonyl-CoA polyketide synthase extender units
Source: Nat Commun. 2016 Dec 21;7:13609. doi: 10.1038/ncomms13609 (PMC5187497; doi:10.1038/ncomms13609)
Supplement: Supplementary Information — Supplementary Figures 1-28, Supplementary Tables 1-4 and Supplementary References. [file ncomms13609-s1.pdf]

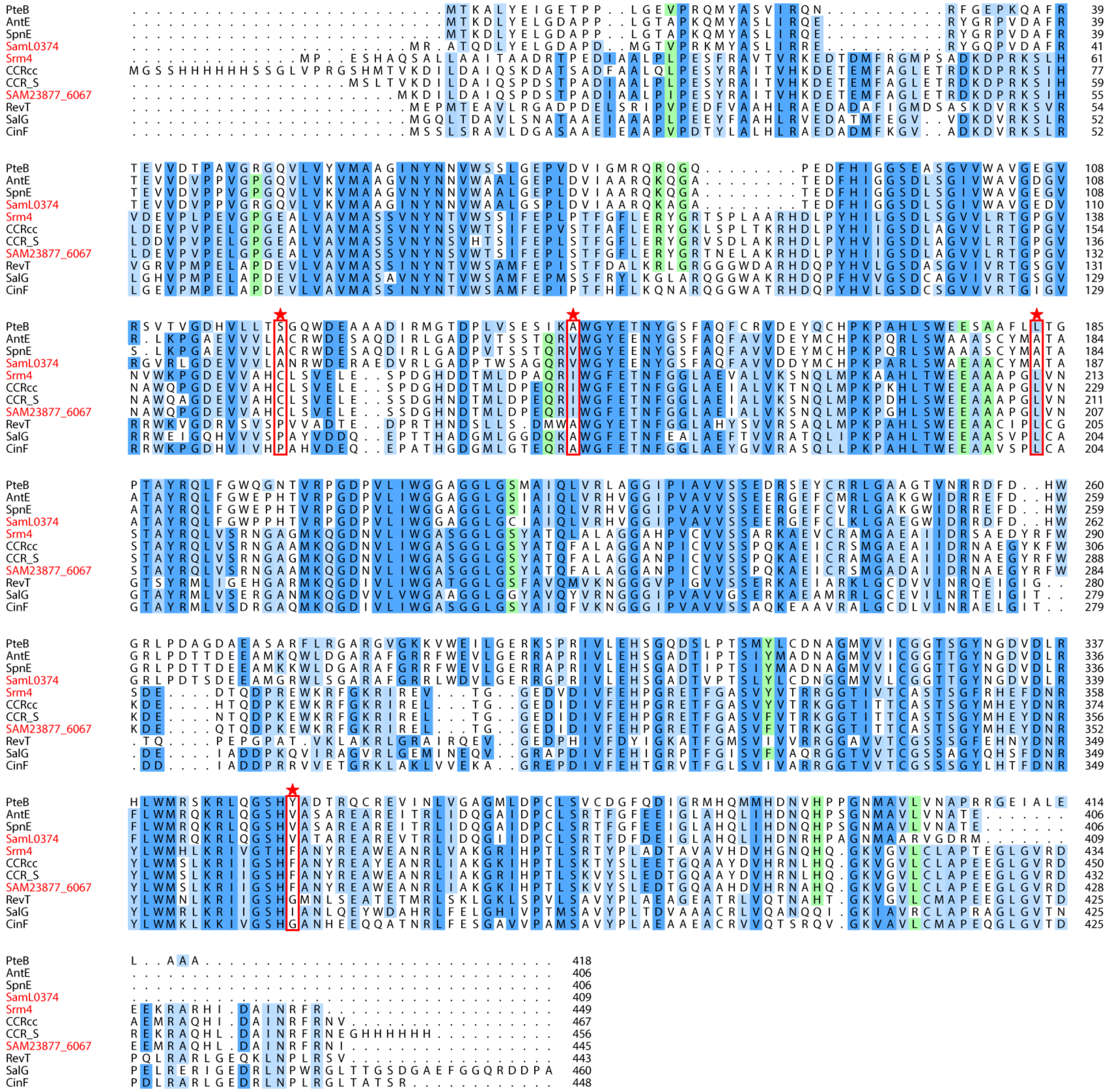

Supplementary Figure 1: **Sequence alignment of CCRC homologues identified in *Streptomyces ambofaciens* ATCC23877 with well characterized CCRCs.** The residues highlighted by the stars and red boxes are essential in defining the substrate specificity of CCRCs.[1-3] The specificity-conferring residues in the CCRC homologues in *S. ambofaciens* ATCC23877 do not resemble those of RevT, PteB, and CinF, all of which are known to be responsible for the biosynthesis of longer alkylmalonyl-CoA extender units. The sequence names highlighted in red are those from *S. ambofaciens* ATCC23877. The sequences designated CCRcc and CCR_S are both specific for crotonyl-CoA and the three CCRC homologues in *S. ambofaciens* have very similar specificity-conferring residues to these enzymes. Accession numbers: CCRcc, 3HZZ; CCR_S, 3KRT; AntE, AGG37751;

SpnE, AKA54628; PteB, WP_010981851; SalG, ABP73651; RevT, BAK64636; CinF, CBW54676; SamL0374 (SAM23877_0426), AKZ53475; Srm4*c (SAM23877_5633), AKZ58678; SAM23877_6076, AK59112

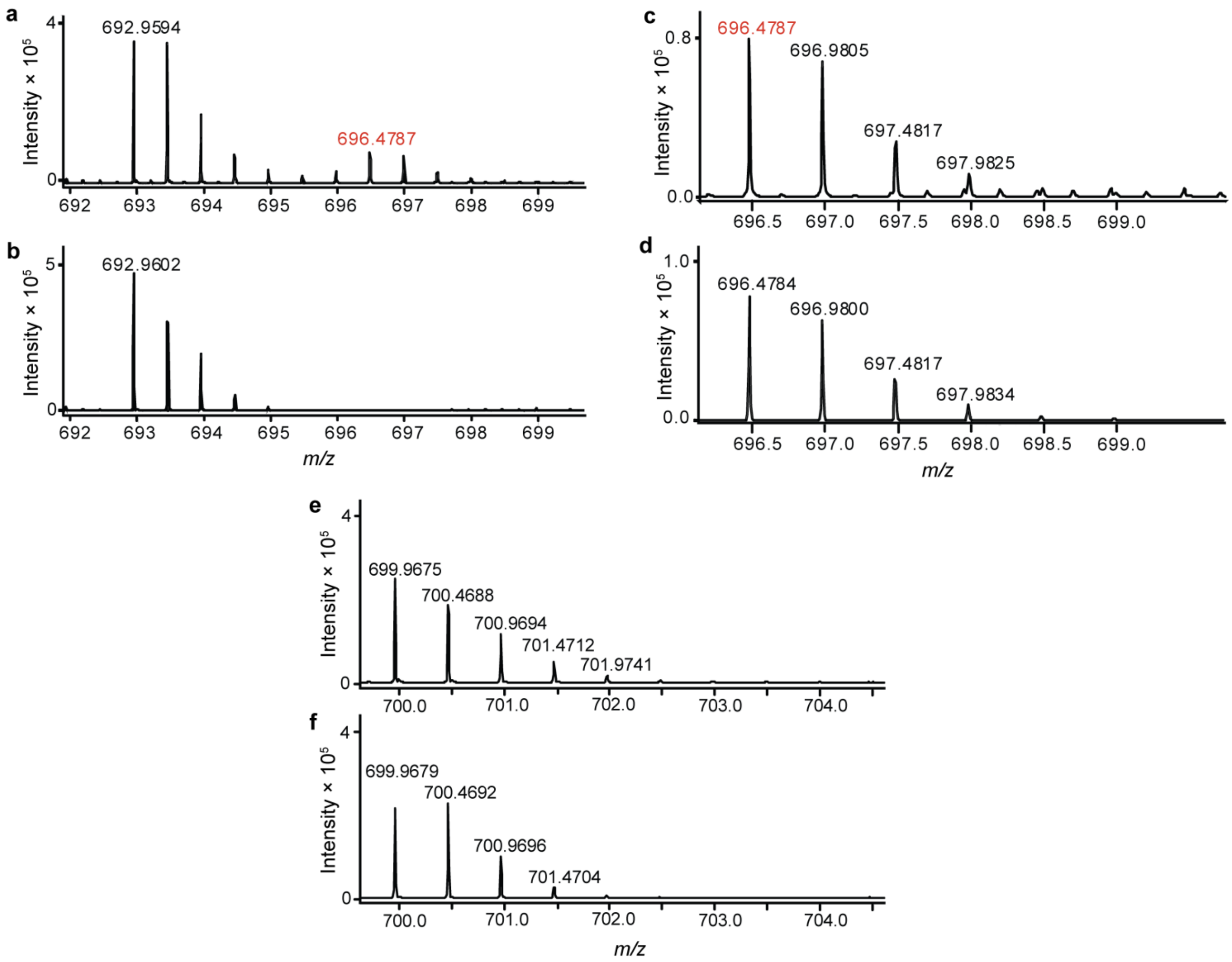

Supplementary Figure 2: **Mass spectra from UHPLC-ESI-Q-TOF-MS analysis of methanolic mycelial extracts from the [U-^{2}H]L-valine 11 incorporation experiments**. Spectra resulting from addition of (**a**) 5 mM [U-^{2}H]L-valine **11** and (**b**) no [U-^{2}H]L-valine to the growth medium. The signal at *m/z* = 692.9594 corresponds to $[M+Na+H]^{2+}$ for stambomycins C/D **3**/**4** and the signal at *m/z* = 696.4787 corresponds to $[M+Na+H]^{2+}$ for stambomycin C **3** derived from incorporation of [U-^{2}H]L-valine **11**. Comparison of the spectrum (**c**) measured for labeled stambomycin C **3** derived from incorporation of [U-^{2}H]L-valine **11** with (**d**) the spectrum calculated for the $[C_{72}H_{125}D_{7}NNaO_{22}]^{2+}$ ion. Spectra for stambomycins A/B **1**/**2** resulting from addition of (**e**) 5 mM [U-^{2}H]L-valine **11** and (**f**) no [U-^{2}H]L-valine to the growth medium. No specific incorporation of the deuterium-labeled precursor is observed.

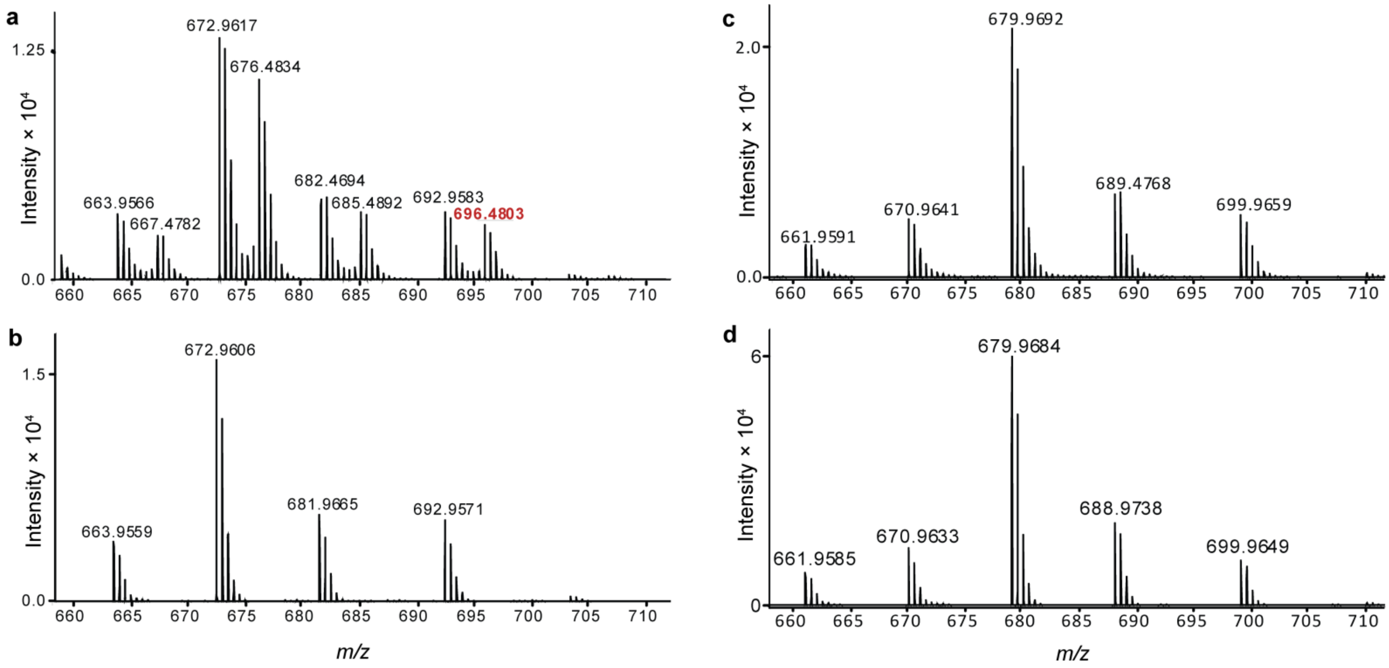

Supplementary Figure 3: **Mass spectra from UHPLC-ESI-Q-TOF-MS analysis of methanolic mycelial extracts from the [U-^{2}H]butyric acid 12 incorporation experiments.** Spectra resulting from addition of (**a**) 5 mM [U-^{2}H]butyric acid **12** and (**b**) no [U-^{2}H]butyric acid to the growth medium. The signal at *m/z* = 692.9583 corresponds to $[M+Na+H]^{2+}$ for stambomycins C/D **3**/**4** and the signal at *m/z* = 696.4803 corresponds to $[M+Na+H]^{2+}$ for stambomycin D **4** derived from incorporation of [U-^{2}H]butyric acid **12** (calculated for $[C_{72}H_{125}D_7NNaO_{22}]^{2+}$:696.4784). Spectra for stambomycins A/B **1**/**2** resulting from addition of (**c**) 5 mM [U-^{2}H]butyric acid **12** and (**d**) no [U-^{2}H]butyric acid to the growth medium. No specific incorporation of the deuterium-labeled precursor is observed.

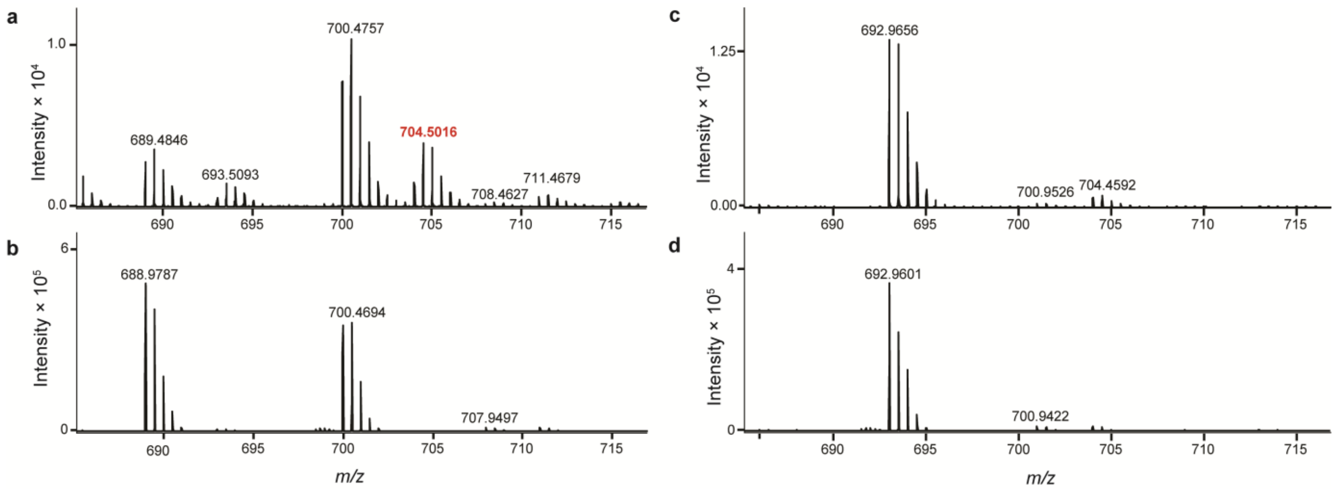

Supplementary Figure 4: **Mass spectra from UHPLC-ESI-Q-TOF-MS analysis of methanolic mycelial extracts from the [U-^{2}H]L-isoleucine 9 incorporation experiments.** Spectra resulting from addition of (**a**) 5 mM [U-^{2}H]L-isoleucine **9** and (**b**) no [U-^{2}H]L-isoleucine to the growth medium. The signal at *m/z* = 700.4757 corresponds to $[M+Na+H]^{2+}$ for stambomycins A/B **1**/**2** and the signal at *m/z* = 704.5016 corresponds to $[M+Na+H]^{2+}$ for stambomycin A **1** derived from incorporation of [U-^{2}H]L-isoleucine **9** (calculated for $[C_{73}H_{125}D_{9}NNaO_{22}]^{2+}$:704.4925;). Spectra for stambomycins C/D **3**/**4** resulting from addition of (**c**) 5 mM [U-^{2}H]L- isoleucine **9** and (**d**) no [U-^{2}H]L-isoleucine to the growth medium. No specific incorporation of the deuterium-labeled precursor is observed.

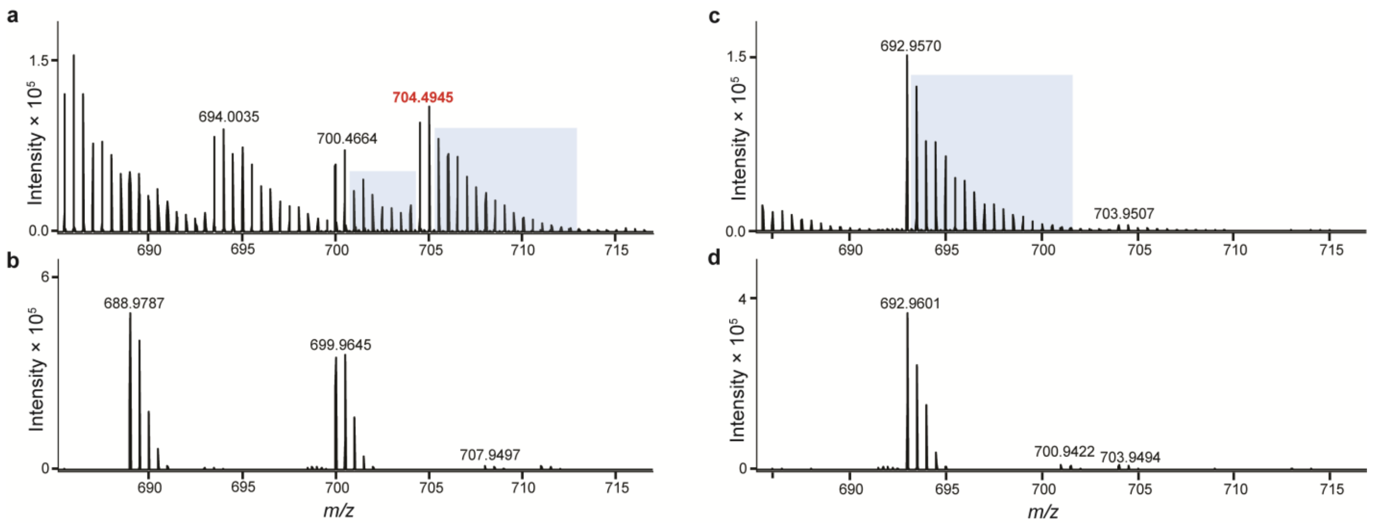

Supplementary Figure 5: **Mass spectra from UHPLC-ESI-Q-TOF-MS analysis of methanolic mycelial extracts from the [U-^{2}H]L-leucine 10 incorporation experiments.** Spectra resulting from addition of (**a**) 5 mM [U-^{2}H]L-leucine **10** and (**b**) no [U-^{2}H]L-leucine to the growth medium. The signal at *m/z* = 699.9645 corresponds to $[M+Na+H]^{2+}$ for stambomycins A/B **1**/**2** and the signal at *m/z* = 704.4945 corresponds to $[M+Na+H]^{2+}$ for stambomycin B **2** derived from incorporation of [U-^{2}H]L-isoleucine **10** (calculated for $[C_{73}H_{125}D_{9}NNaO_{22}]^{2+}$:704.4925). Spectra for stambomycins C/D **3**/**4** resulting from addition of (**c**) 5 mM [U-^{2}H]L-leucine **10** and (**d**) no [U-^{2}H]L-leucine to the growth medium. No specific incorporation of the deuterium-labeled precursor was observed. The regions of the spectra highlighted by the blue boxes result from the incorporation of deuterium labeled acetyl-CoA derived from the catabolism of [U-^{2}H]L-leucine **10**.

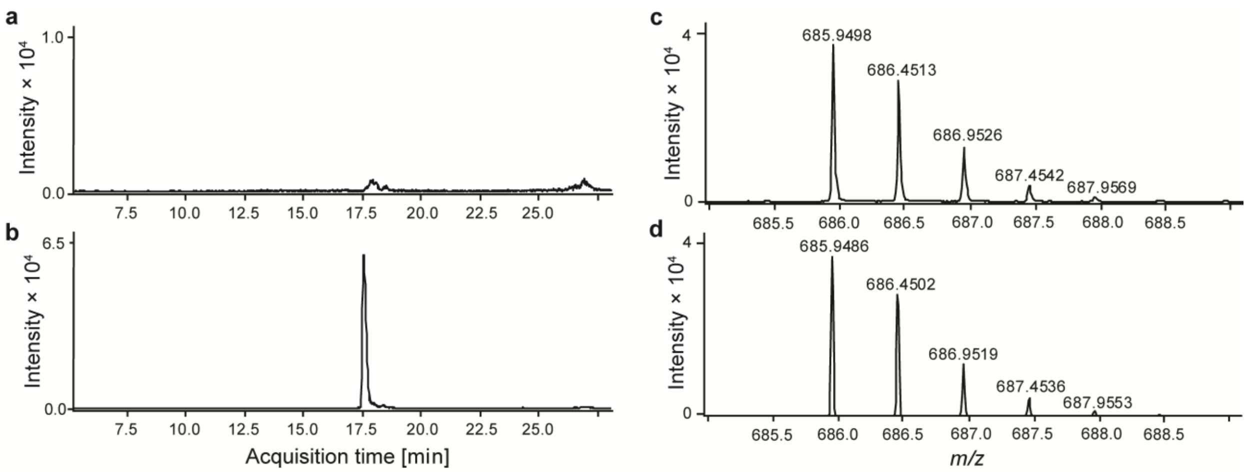

Supplementary Figure 6: **UHPLC-ESI-Q-TOF-MS analysis of methanolic mycelial extracts from the *n*-heptanoic acid incorporation experiments.** Extracted ion chromatogram at *m/z* = 685.9, corresponding to $[M+Na+H]^{2+}$ for stambomycin analogue **22**, from UHPLC-ESI-Q-TOF-MS analyses of methanolic mycelial extracts *S. ambofaciens* W130 grown in the absence (**a**) and (**b**) presence of 5 mM *n*-heptanoic acid. (**c**) Mass spectrum of **22** from UHPLC-ESI-Q-TOF-MS analyses of *S. ambofaciens* W130 fed with *n*-heptanoic acid. (**d**) Simulated spectrum for $C_{71}H_{130}NNaO_{22}^{2+}$.

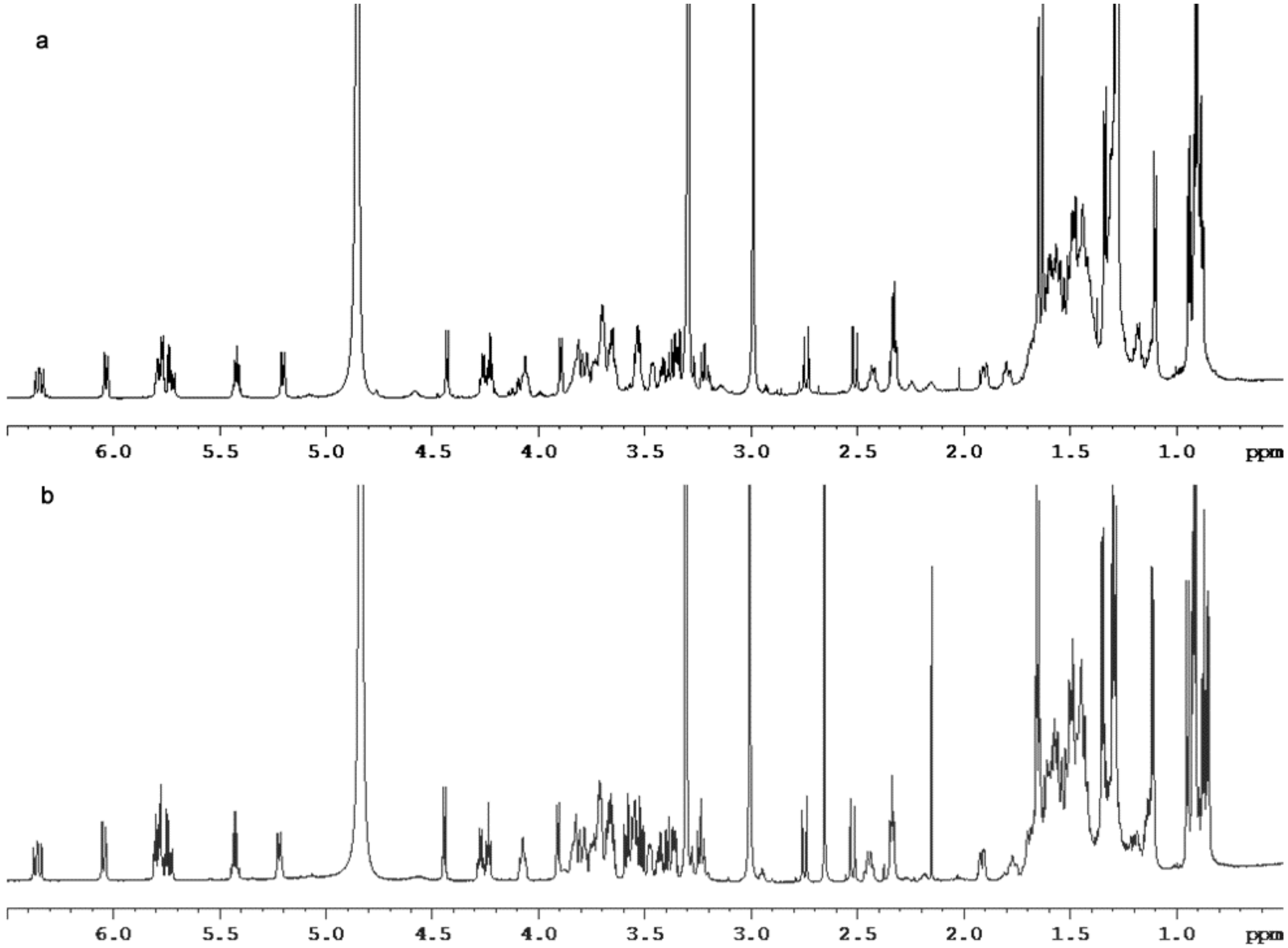

Supplementary Figure 7: **Comparison of the ^{1}H NMR spectra (700 MHz, d_4-MeOH) of (a) 22 and (b) stambomycins C/D 3/4.**

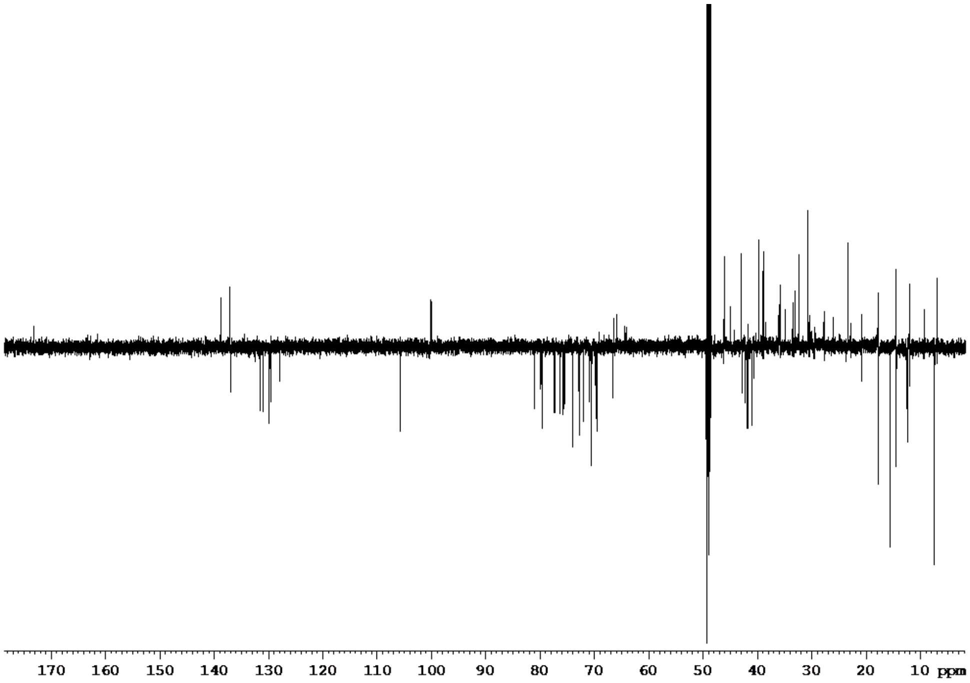

Supplementary Figure 8: **^{13}C-APT NMR spectrum (175 MHz, d_4-MeOH) of 22.**

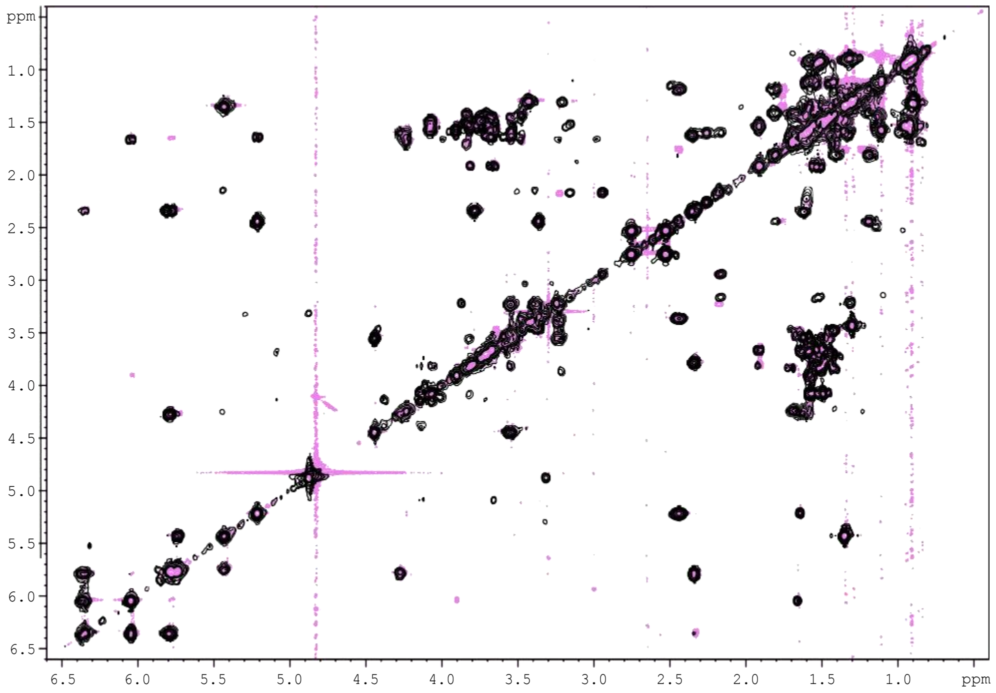

Supplementary Figure 9: **Overlaid COSY NMR spectra (700 MHz, d_4-MeOH) of 22 (black) and stambomycins C/D 3/4 (pink).**

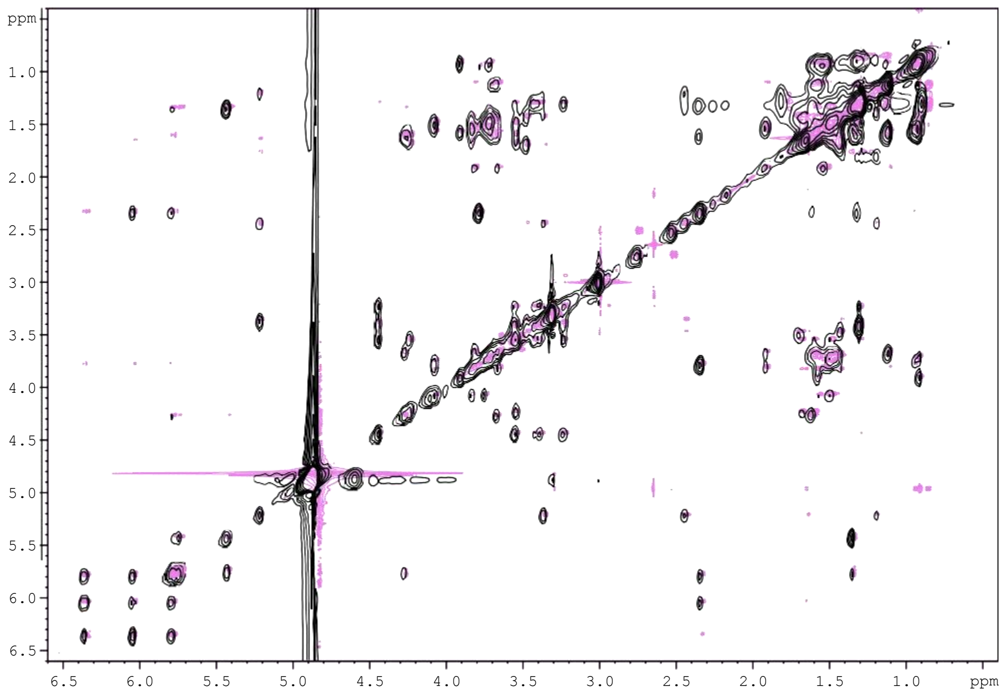

Supplementary Figure 10: **Overlaid TOCSY NMR spectra (700 MHz, d_4-MeOH) of 22 (black) and stambomycins C/D 3/4 (pink).**

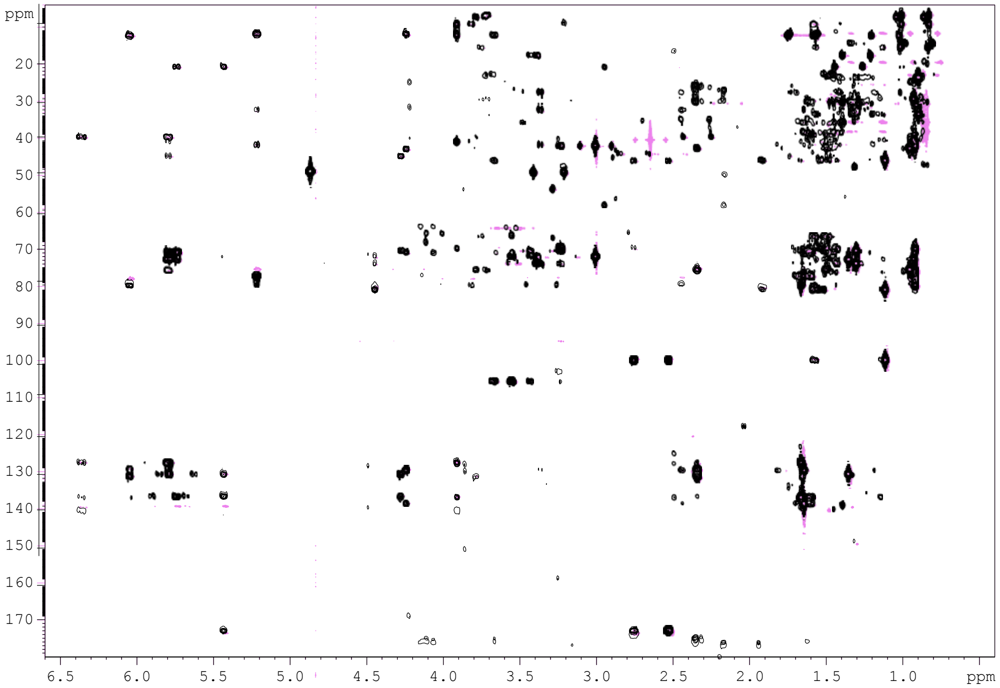

Supplementary Figure 11: **Overlaid HMBC NMR spectra (700 MHz / 175 MHz, d4-MeOH) of 22 (black) and stambomycins C/D 3/4 (pink).**

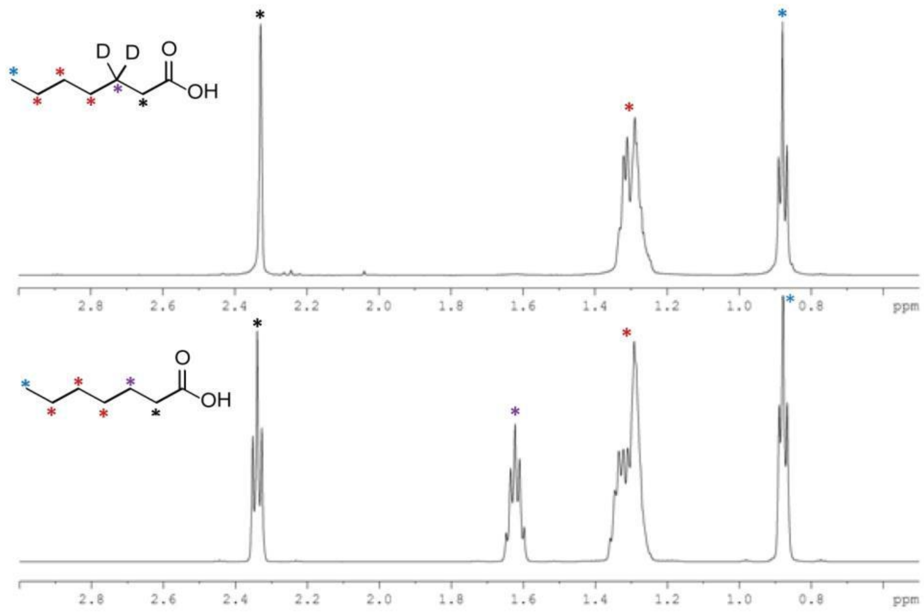

Supplementary Figure 12: **Comparison of the ^{1}H NMR spectra of [3-$^{2}H_{2}$]heptanoic acid 23 and unlabeled *n*-heptanoic acid 21.**

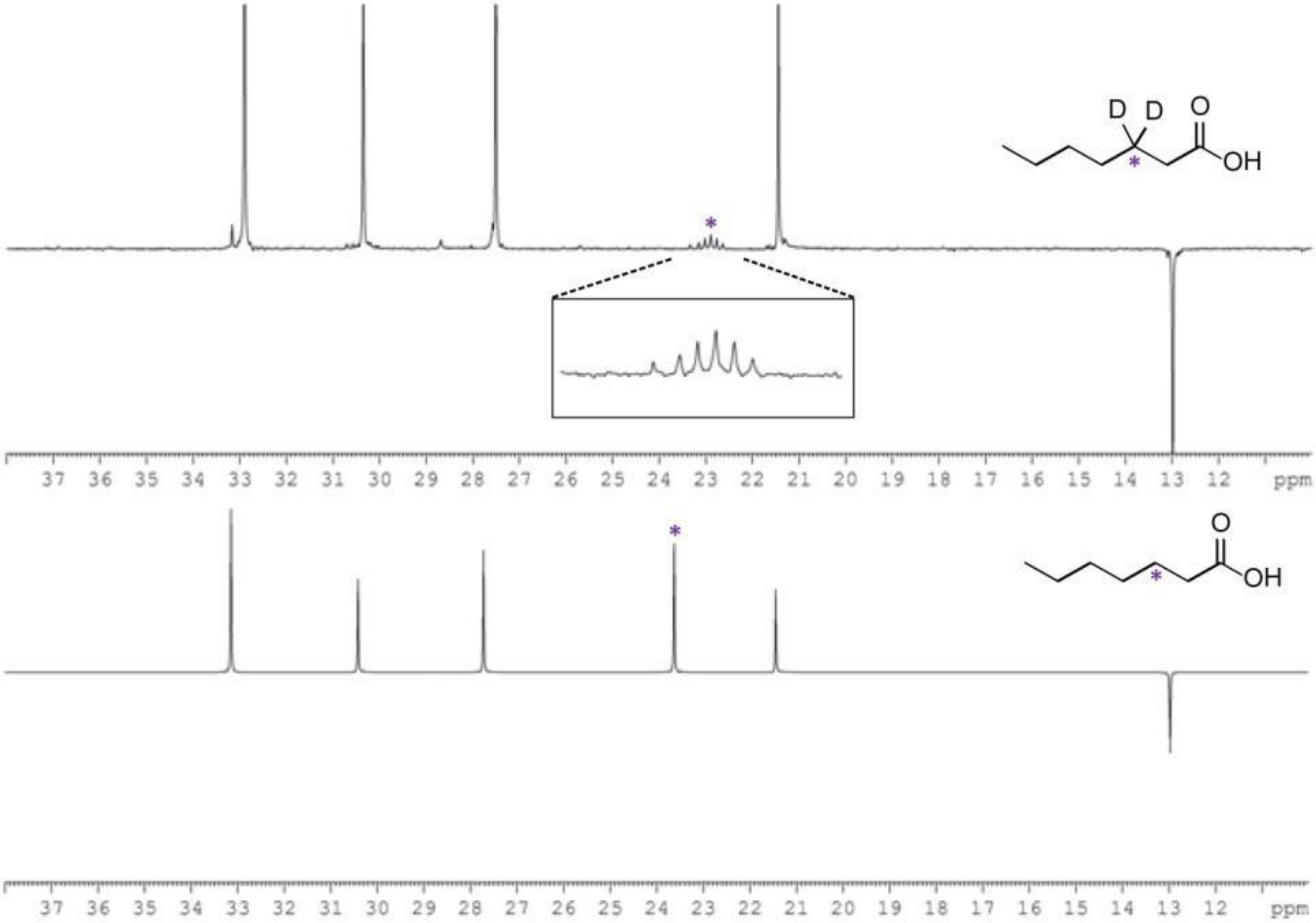

Supplementary Figure 13: **Comparison of the ^{13}C NMR spectra of [3-$^{2}H_{2}$]heptanoic acid 23 and unlabelled *n*- heptanoic acid 21**. The splitting of the C-3 signal as a result of the attached deuterium atoms is shown in the inset.

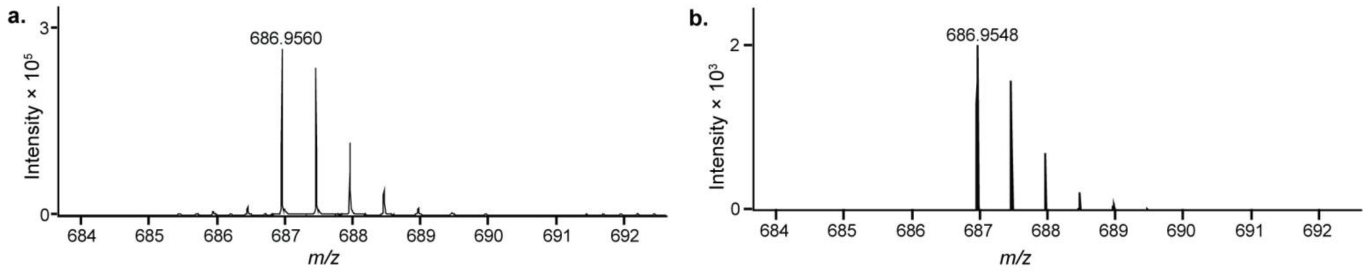

Supplementary Figure 14: **Mass spectra from UHPLC-ESI-Q-TOF-MS analysis of the stambomycin analogue 22 produced as a result of feeding [3-$^{2}H_2$]heptanoic acid to *S. ambofaciens* W130.** (**a**) Measured spectrum. (**b**) Simulated spectrum for $C_{71}H_{128}D_2NNaO_{22}^{2+}$ corresponding to $[M+Na+H]^{+}$ for doubly labeled **22**.

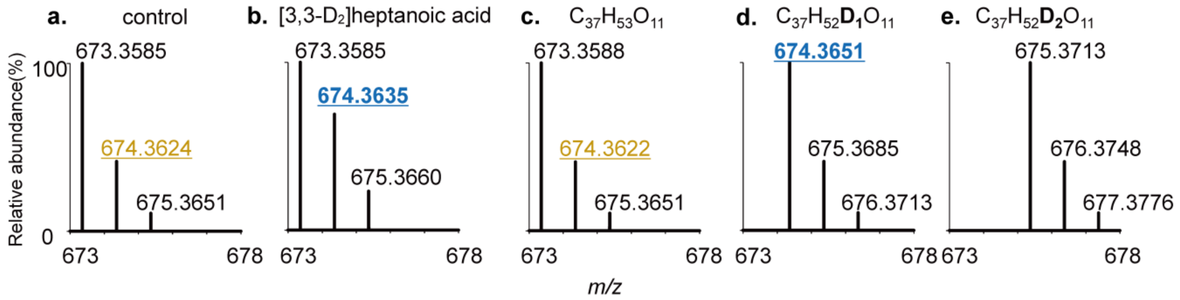

Supplementary Figure 15: Mass spectra from ESI-TOF-MS analysis of partially purified reveromycin D 32 produced from [3-$^{2}H_2$]heptanoic acid incorporation experiments. Comparison of ESI-TOF mass spectra of partially purified reveromycin D **32** from cultures of *Streptomyces* sp. SN-593 Δ*revR* mutant grown in the absence (**a**) and presence (**b**) of [3-$^{2}H_2$]heptanoic acid. Simulated isotope distributions for unlabeled (**c**), singly-labeled (**d**) and doubly-labeled reveromycin D (**e**). The data are consistent with 20% incorporation of singly-labeled heptanoic acid into **32**.

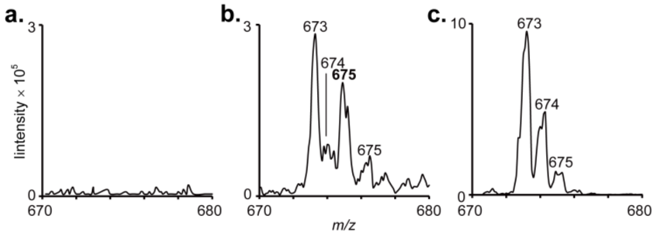

Supplementary Figure 16: LC-MS analysis of partially purified extracts from the [3-$^{2}H_2$]heptanoic acid incorporation experiments. Mass spectra of reveromycin D **32** from LC-MS analyses of partially purified extracts from the cultures of (**a**) the Δ*revT* mutant, (**b**) the Δ*revT::samR0483* mutant to which [3-$^{2}H_2$]heptanoic acid **23** has been fed, and (**c**) wild type *Streptomyces* sp. SN-593.

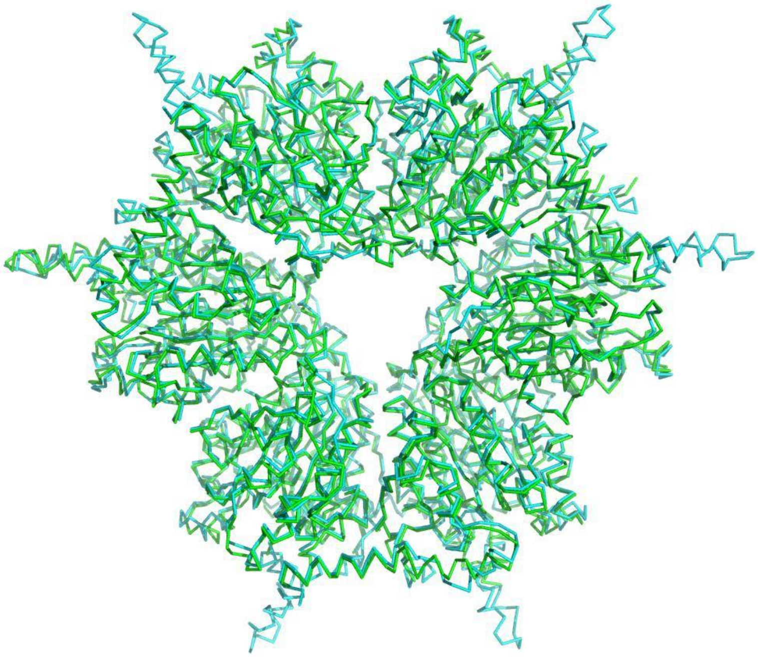

Supplementary Figure 17: Superimposition of PccB from *S. coelicolor* (cyan) with MccB from *S. ambofaciens* (green). The structures are displayed as colored ribbons.

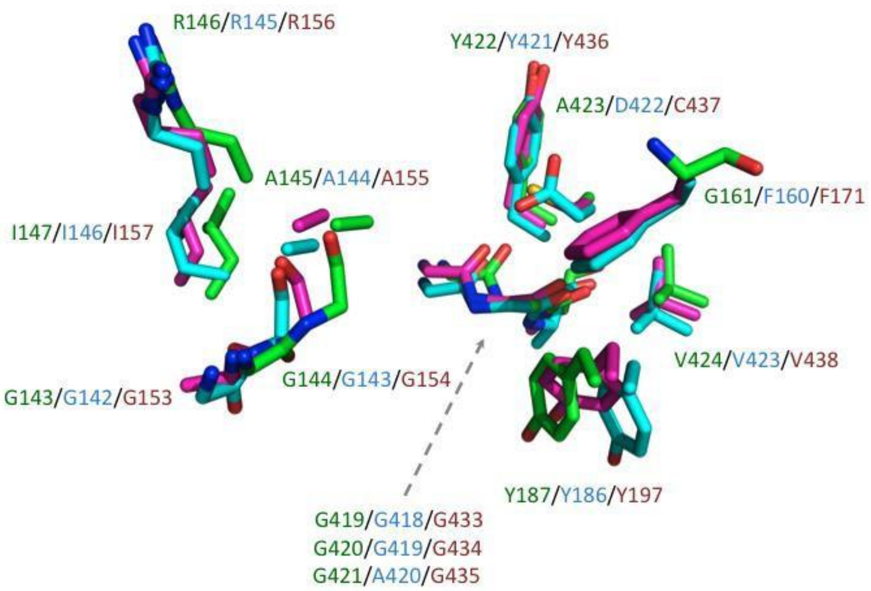

Supplementary Figure 18: Structural comparison of MccB (PDB ID: 5INI), PccB (PDB ID: 1XNY), and AccD5 (PDB ID: 2A7S) active site residues. Colors are green, cyan, and magenta, respectively.

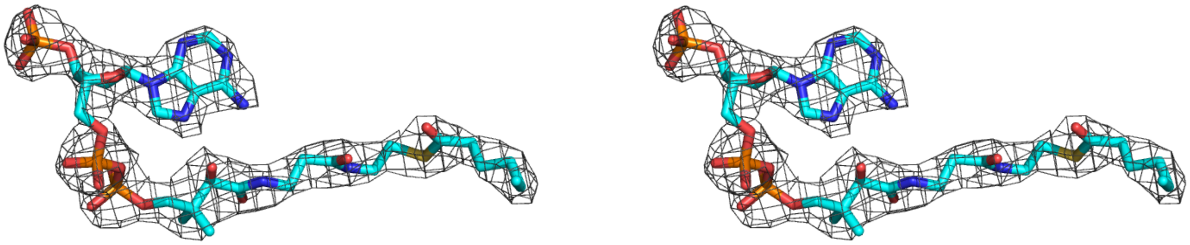

Supplementary Figure 19: **Stereo view of hexanoyl-CoA bound to MccB with 2F0-FC SA omit map contoured 1.0 σ at 2.85 Å.** The surrounding protein has been removed for clarity.

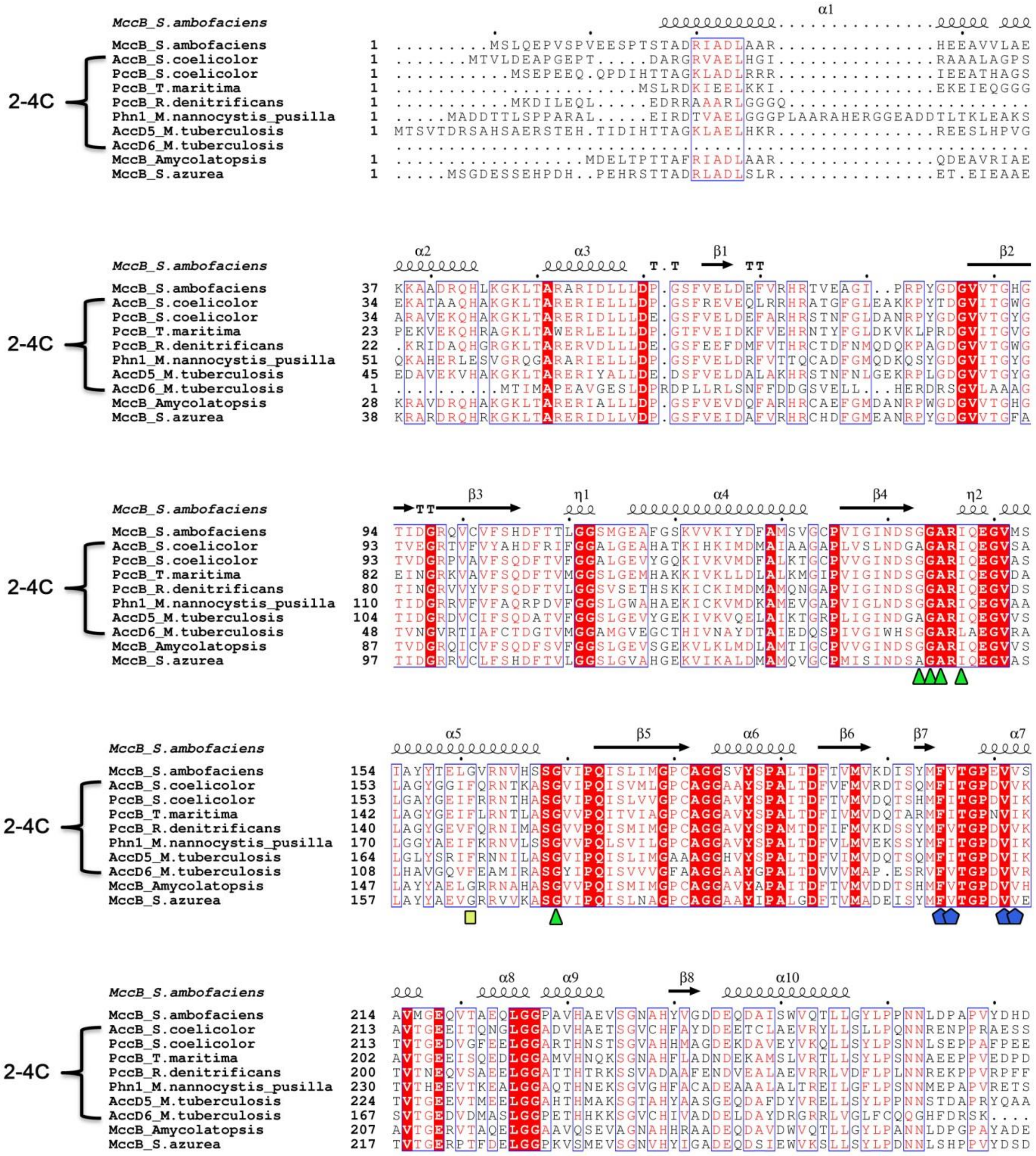
MccB_S.ambofaciens
MccB_S.ambofaciens
AccB_S.coelicolor
PccB_S.coelicolor
PccB_T.maritima
PccB_R.denitrificans
Phn1_M.nannocystis_pusilla
AccD5_M.tuberculosis
AccD6_M.tuberculosis
MccB_Amycolatopsis
MccB_S.azurea
2-4C
α1
α2
α3
β1
β2
β3
η1
α4
β4
η2
α5
β5
α6
β6
β7
α7
α8
α9
β8
α10

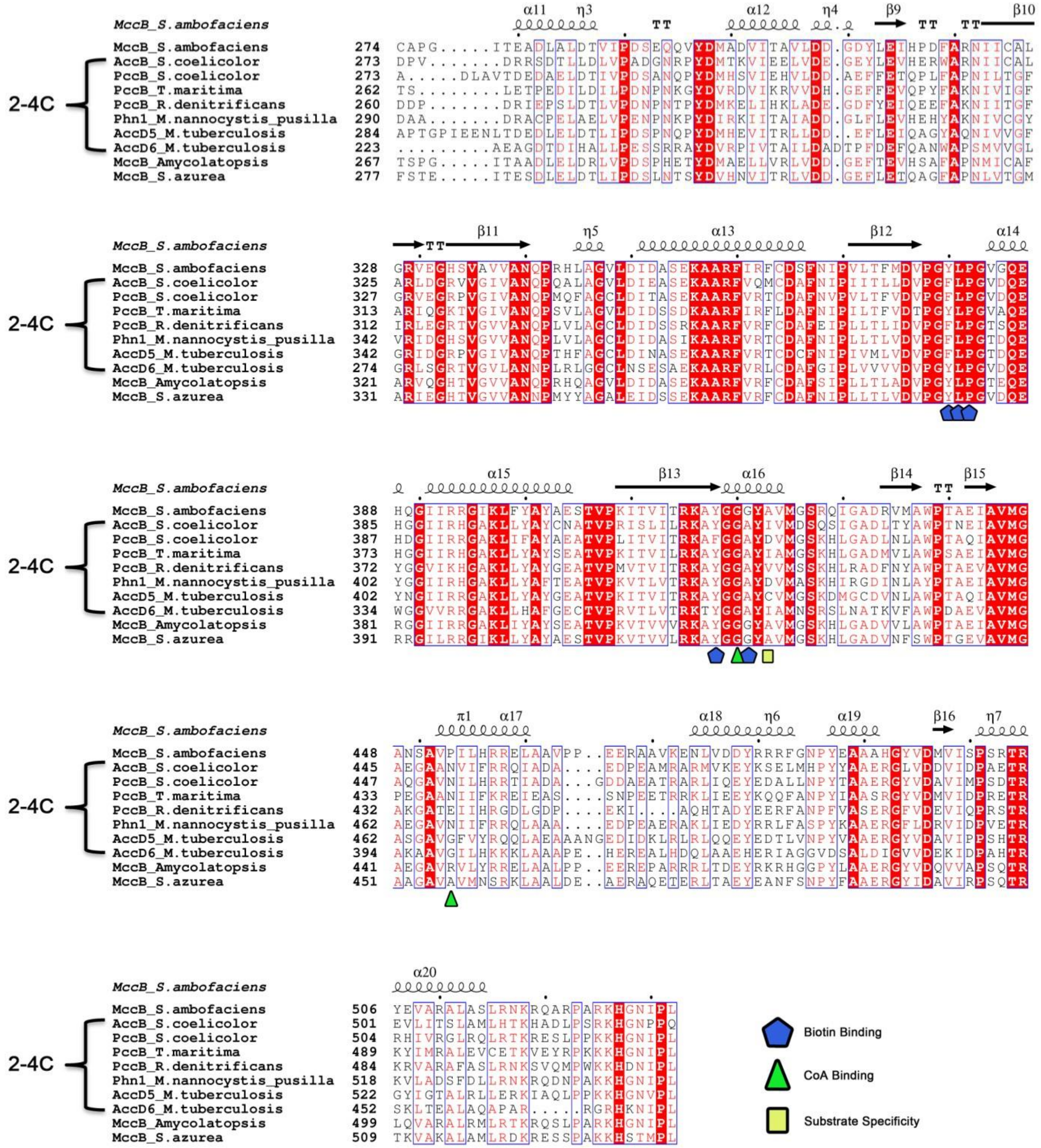

Supplementary Figure 20: **Sequence alignment of MccB from *S. ambofaciens* with other ACC β-subunits**. The enzymes denoted 2-4C use acetyl-, propionyl or butyryl-CoA as a substrate. MccB in *Streptomyces azurea* isproposed to assemble butylmalonyl-CoA and other 6-8 carbon extender units incorporated by PriA6 into the primycins.[9]

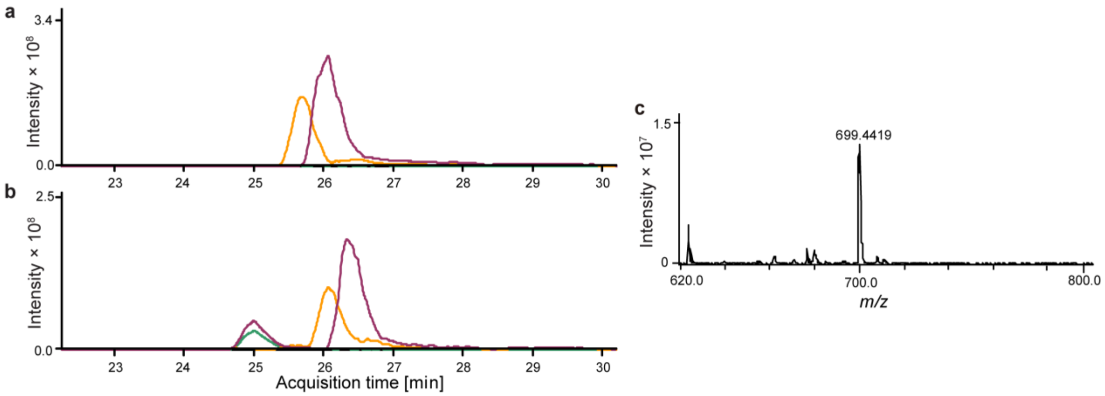

Supplementary Figure 21: **UHPLC-ESI-Q-TOF-MS analysis of methanolic mycelial extracts from the 6-azidohexanoic acid incorporation experiments.** EICs at *m/z* = 699.4, corresponding to $[M+H+Na]^{2+}$ for stambomycin analogue **37** (green), at *m/z* = 692.9, corresponding to $[M+H+Na]^{2+}$ for stambomycins C/D **3**/**4** (orange), and at *m/z* = 699.9, corresponding to $[M+H+Na]^{2+}$ for stambomycins A/B **1/2** (purple), from UHPLC-ESI-Q-TOF-MS analyses of methanolic mycelial extracts of *S. ambofaciens* W130 grown in the absence (**a**) and presence (**b**) of 5 mM 6-azidohexanoic acid. (**c**) Measured mass spectrum for peak corresponding to stambomycin analogue **37** with a retention time of 25 minutes (calculated for $C_{70}H_{127}N_4NaO_{22}^+$: 699.4414).

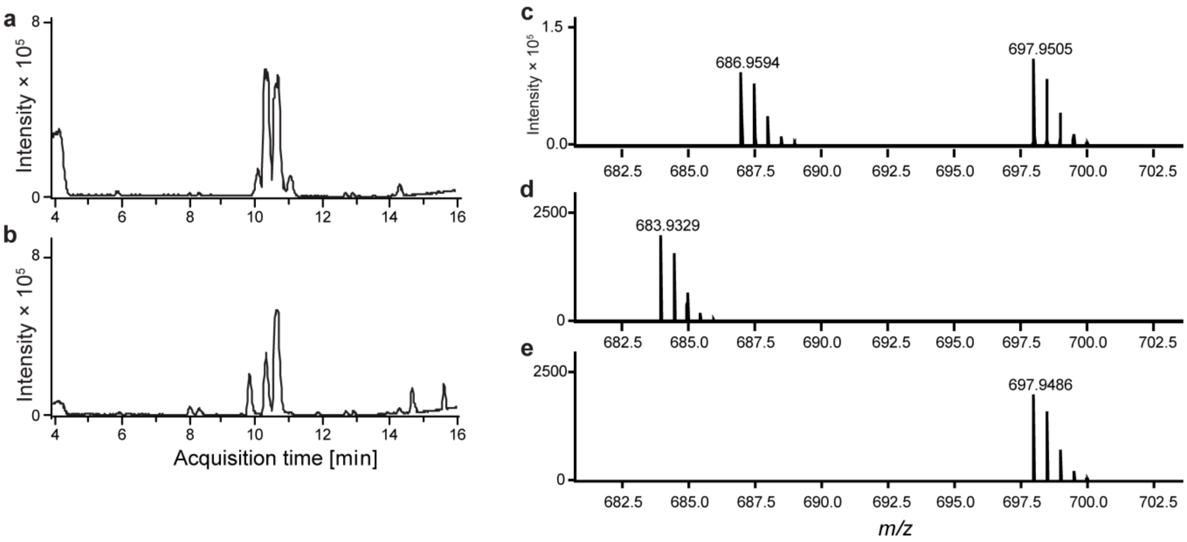

Supplementary Figure 22: **UHPLC-ESI-Q-TOF-MS analysis of methanolic mycelial extracts from the 6-heptynoic acid incorporation experiments.** Base peak chromatograms from UHPLC-ESI-Q-TOF-MS analyses of mycelial extracts of *S. ambofaciens* W130 grown in the absence (**a**) and presence (**b**) of 5 mM 6-heptynoic acid. The peaks with retention times of 10.3 minutes and 10.7 minutes correspond to stambomycins C/D **3/4** and stambomycins A/B **1/2**, respectively. A new peak with a retention time of 9.8 minutes, corresponding to a novel stambomycin analogue is observed. (**c**) Mass spectrum of the stambomycin analogue with a retention time of 9.8 minutes observed in UHPLC-ESI-Q-TOF-MS analyses of *S. ambofaciens* W130 fed with 6-heptynoic acid. (**d**) Simulated spectrum for $C_{71}H_{126}NNaO_{22}^{2+}$, corresponding to the stambomycin analogue expected to result from direct incorporation of 6-heptynoic acid. (**e**) Simulated spectrum for $C_{73}H_{130}NNaO_{22}^{2+}$, corresponding to stambomycin analogue **39**.

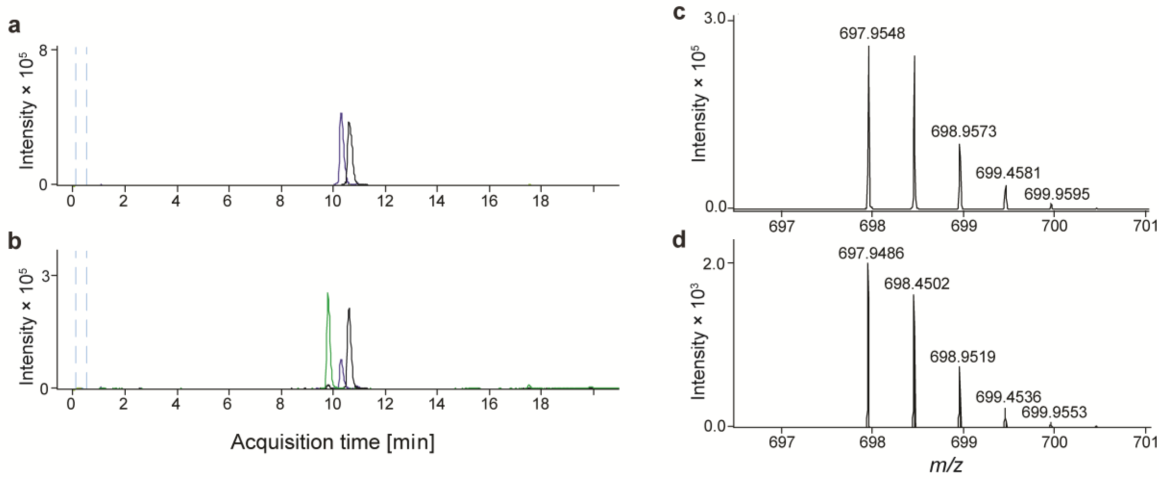

Supplementary Figure 23: **UHPLC-ESI-Q-TOF-MS analysis of methanolic mycelial extracts from the 8-nonynoic acid incorporation experiments.** EICs at *m/z* = 697.9, corresponding to $[M+H+Na]^{2+}$ for stambomycin analogue **39** (green), at *m/z* = 692.9, corresponding to $[M+H+Na]^{2+}$ for stambomycins C/D **3**/**4** (blue), and at *m/z* = 699.9, corresponding to $[M+H+Na]^{2+}$ for stambomycins A/B **1/2** (black), from UHPLC-ESI-Q-TOF-MS analyses of methanolic mycelial extracts of *S. ambofaciens* W130 grown in the absence (**a**) and presence (**b**) of 5 mM 8-nonynoic acid. (**c**) Measured and (**d**) simulated mass spectra for the $[M+Na+H]^{2+}$ ion of stambomycin analogue **39**.

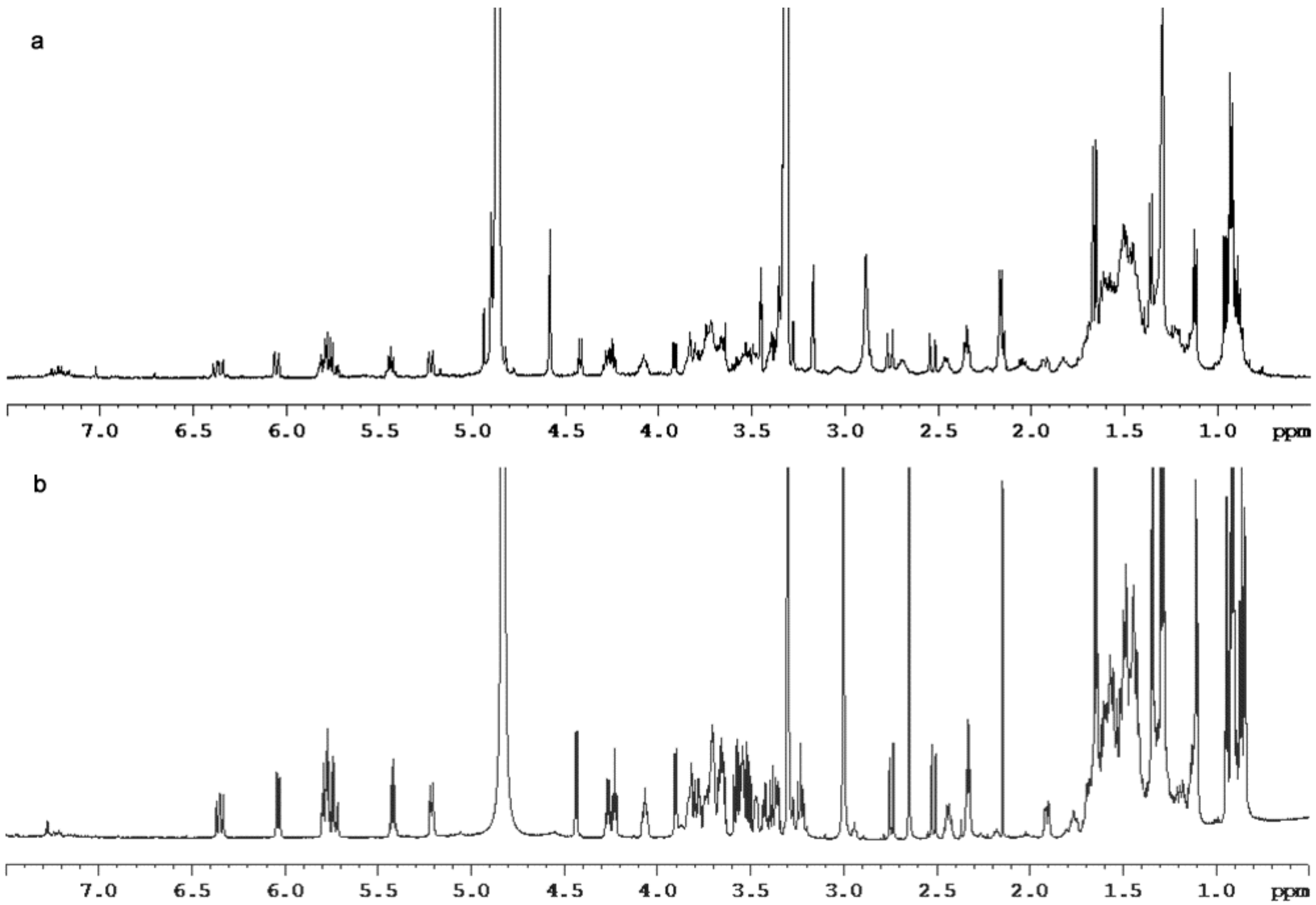

Supplementary Figure 24: **Comparison of ^{1}H-NMR spectra (600 MHz, MeOD) for (a) stambomycin analogue 39 and (b) stambomycins C/D 3/4 .**

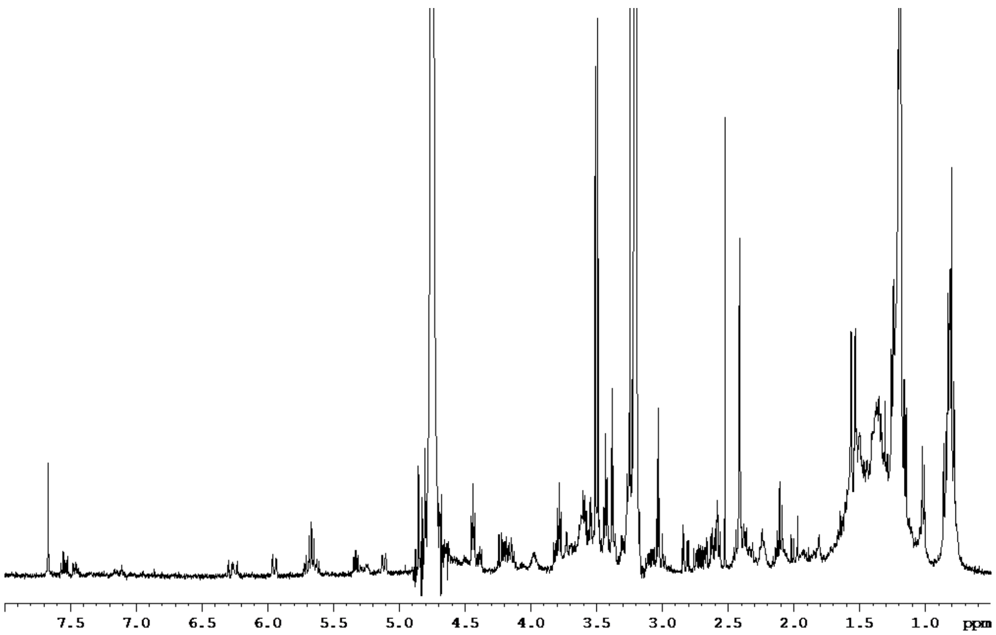

Supplementary Figure 25: **^{1}H-NMR (400 MHz, MeOD) spectrum of biotinylated stambomycin analogue derived from the click reaction of stambomycin analogue 39 with the azide-PEG3-biotin conjugate**. The signal at 7.68 ppm corresponds to the triazole proton and the signals at 4.2 ppm correspond to the protons around the fused thiolane ring of biotin.

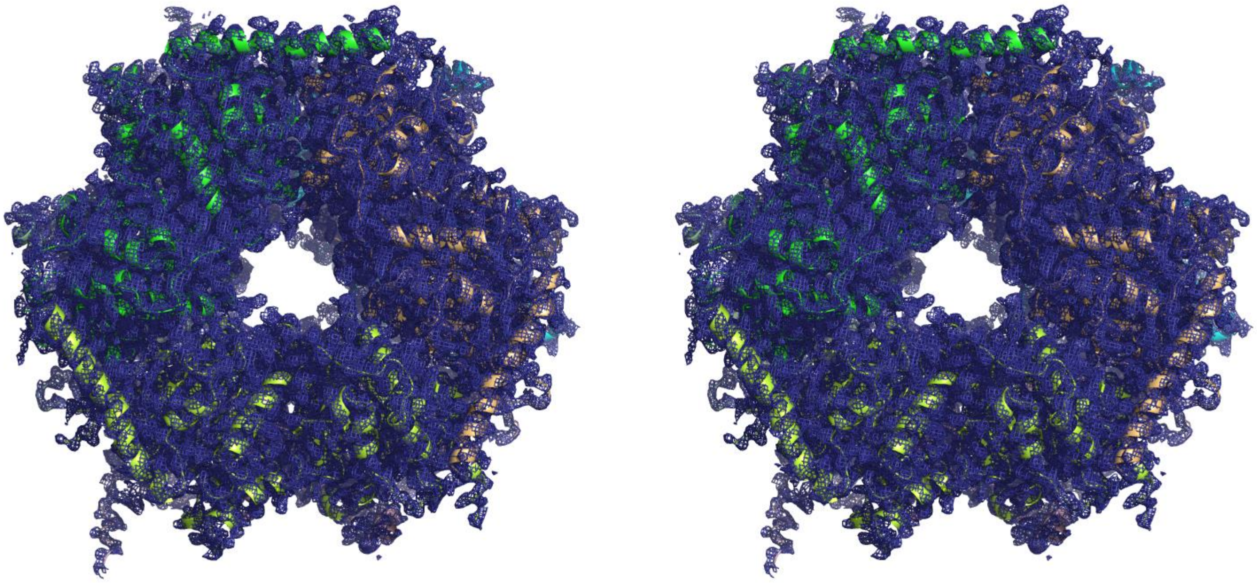

Supplementary Figure 26: Stereo view of the apo-MccB hexamer with 2F0-FC map contoured to 1.0 σ at 2.45 Å.

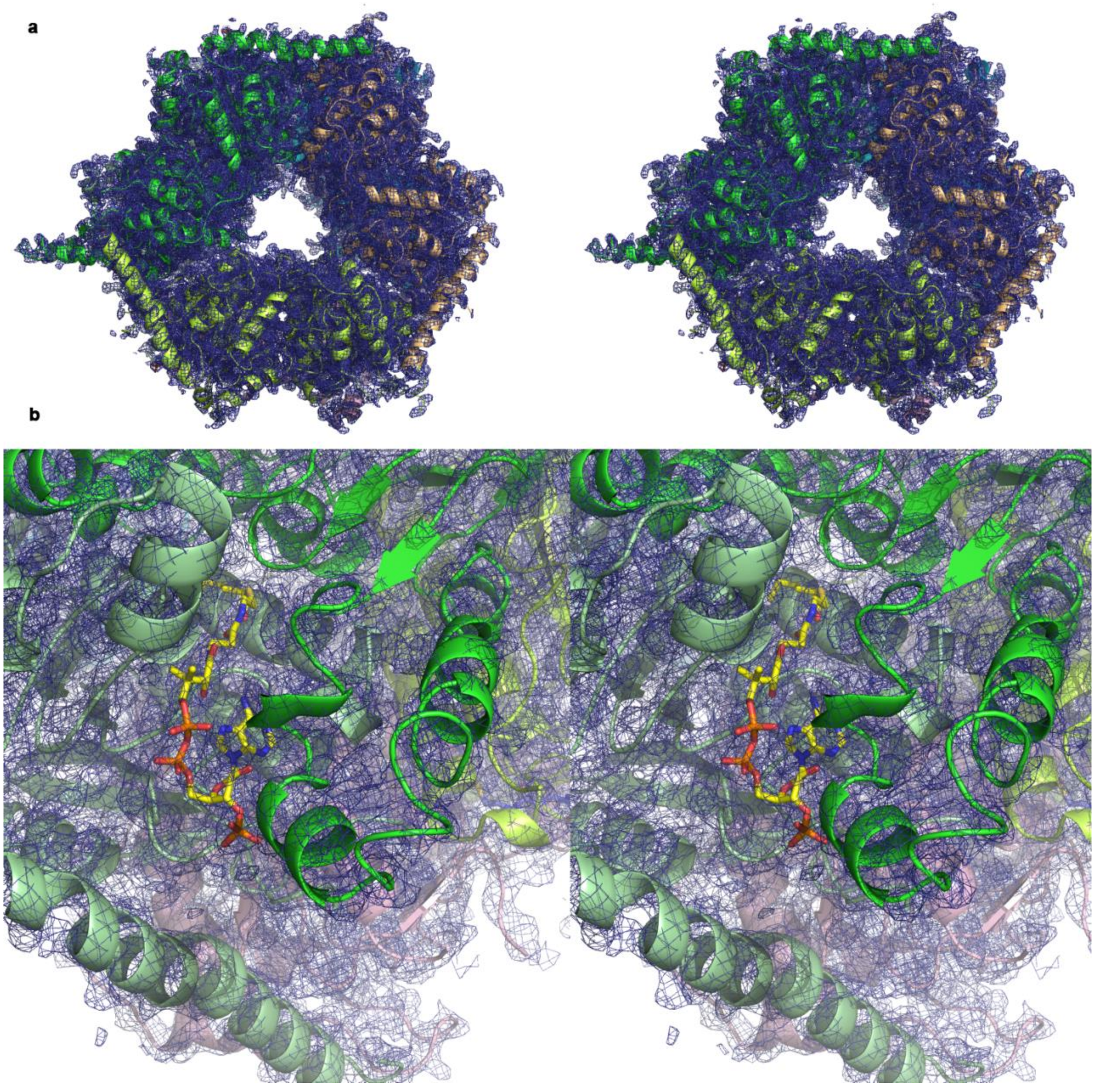

Supplementary Figure 27: Stereo views of the MccB hexamer bound to four molecules of hexanoyl-CoA. (**a**) The overall architecture of MccB bound to four molecules of hexanoyl-CoA. (**b**) The active site pocket at the dimer interface of opposing MccB monomers. 2F0-FC maps are contoured to 1.0 σ at 2.85 Å.

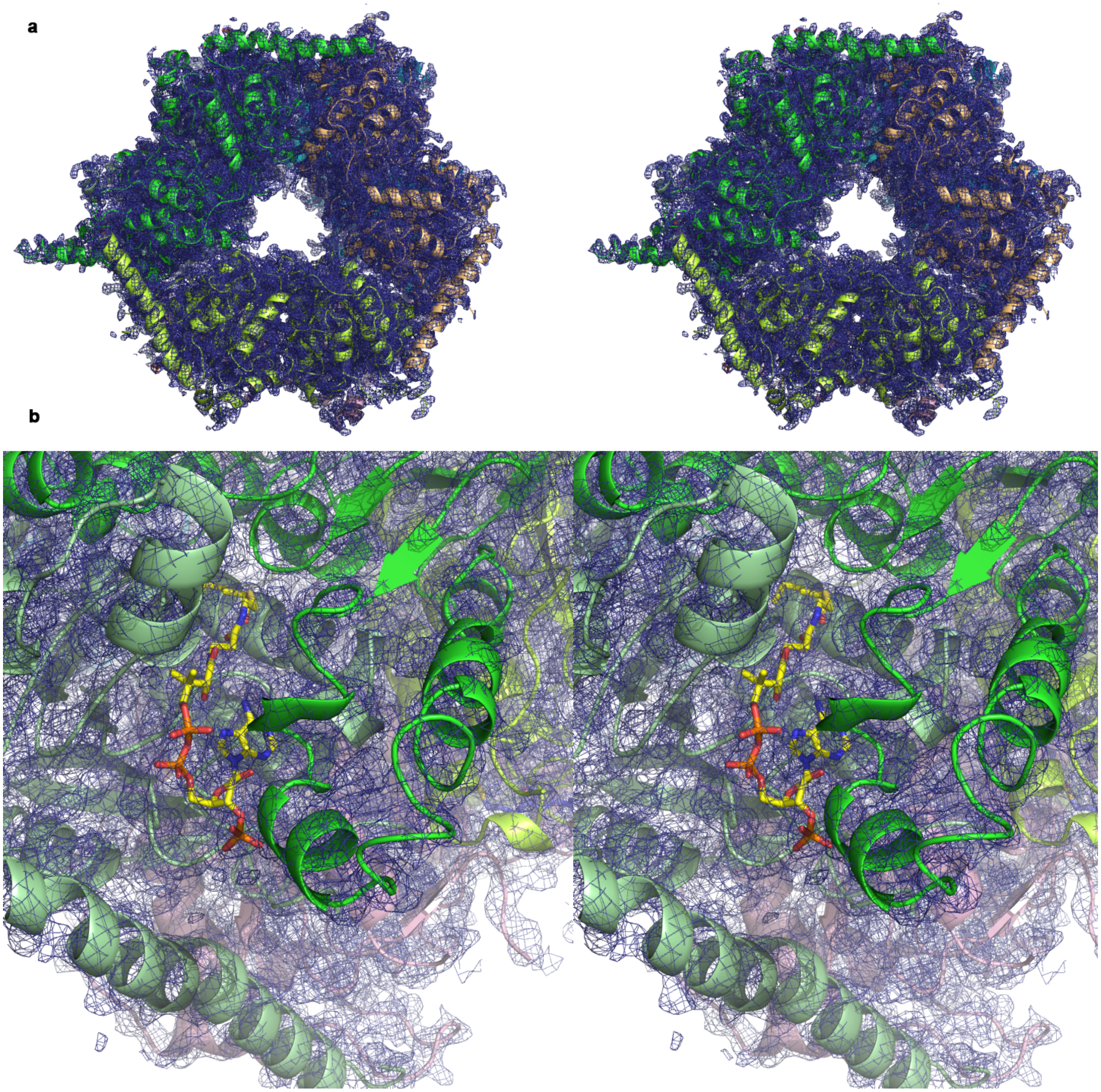

Supplementary Figure 28: **Stereo views of the MccB hexamer bound to one molecule of hexanoyl-CoA.** (**a**) The overall architecture of MccB bound to one molecule of hexanoyl-CoA. (**b**) The active site pocket at the dimer interface of opposing MccB monomers. 2F0-FC maps are contoured to 1.0 σ at 2.75 Å.

Supplementary Table 1: Bacterial strains and plasmids

Strain/plasmid	Relevant characteristics	Ref.
**Strain**		
S. ambofaciens ATCC 23877	Wild-type strain, in which stambomycin cluster is poorly expressed	4
S. ambofaciens W130	Stambomycin producing strain in which *samR0484* is integrated into the chromosome and is under control of the *erm*E* promoter	This work
E. coli ET12567/pUZ8002	Strain used for conjugal transfer of pOSV556t derivatives into *S. ambofaciens* ATCC 23877	5
Streptomyces sp. SN-593	Wild-type reveromycin-producing strain	6
E. coli GM2929 *hsdS::* Tn10 / pUB307-*aph*::Tn*7*	Strain used for conjugal transfer of pTYM19 derivatives into *Streptomyces sp.* SN-593	7
Δ*revT*	*revT* disruptant of *Streptomyces* sp. SN-593	8
Δ*revR*	*revR* disruptant of *Streptomyces* sp. SN-593	8
Δ*revT::samR0483*	Δ*revT* mutant complemented with pTYM19-P_{aph}-*samR0483*	This work
**Plasmid**		
pOSV556t	Integrative *E. coli*-*Streptomyces* shuttle vector	9
pOSV556t-*samR0484*	*samR0484* inserted between the *Hin*dIII and *Cla*I sites of pOSV556t	This work
pOSV556t-*samR0483*	*samR0483* inserted between the *Hin*dIII and *Cla*I sites of pOSV556t	This work
pTYM19	Integrative *Streptomyces*-*E. coli* shuttle vector	10
pTYM19-P_{aph}	Derivative of pTYM19 in which the *aphII* promoter fragment is inserted into the *Eco*RI and *Bam*HI sites.	11
pTYM19-P_{aph}-*samR0483*	*samR0483* inserted into *Bam*HI and *Hind*III sites of pTYM19-P_{aph}	This work
pET151-*samR0483*	MccB overproduction construct	This work
pET28a-*samR0483*	MccB overproduction construct	This work

Supplementary Table 2: Oligonucleotide primers

Target gene (template)	Primers	Ref.
samR0484 (for cloning into pOSV556t between *Hind*III and *Cla*I)	pOSV_samR0484_FW: 5'-AAAGGGAAGCTTAGGAGGCCAGTCATTGCTGGTCC-3'<br>pOSV_samR0484_RV: 5'-CCCTTTATCGATCGTGGGCAGGCTCTGCTC-3'	This work
samR0483 (for cloning into pOSV556t between *Hind*III and *Cla*I)	pOSV_samR0483_FW: 5'-AAGGGAAGCTTGCTGGTATGTCGTCCCA-3'<br>pOSV_samR0483_RV: 5'- CCCTTTATCGATACGACGTCACA -3'	This work
samR0483 (pOSV556t-*samR0483*)	0483_BamHI: 5'-CGCGGATCCATGTCGCTCCAGGA-3'<br>0483_HindIII: 5'-GGGAAGCTTTCACAGGGGAATGT-3'	This work
samR0483 (For TOPO cloning into pET151)	TOPO_483_FW: 5'-CACCATGTCGCTCCAGGAGCCTGTCTC-3'<br>TOPO_483_R: 5'-TCACAGGGGAATGTTCCCGTGTT-3'	This work
samR0483 in pET151 (For cloning into pET28a between *Nhe*I and *Not*I)	pET28a-samR0483_FW: 5'- ATGCGCGCTAGCGATTCTACGGAAAAC-3'<br>pET28a-samR0483_RV: 5'-TATATAGCGGCCGCTCACAGGGGAATGTT-3'	This work

Supplementary Table 3: Comparison of ^{1}H and ^{13}C NMR data for stambomycin D[1] (**4**) and stambomycin analogue **22**. The atom numbering is shown in Figure 3 of the main manuscript.

Position	δ_C (ppm) in 4	δ_C (ppm) in 22	δ_H (ppm) in 4	δ_H (ppm) in 22	J_{H-H} (Hz)
1	173.3	173.1	-	-	
2	44.3	44.3	2.52, 2.83	2.53, 2.76	14.5
3	100.0	100.1	-	-	
4	46.2	46.2	1.56	1.57	
4-Me	12.4	12.5	1.04	1.11	
5	81.0	81.0	3.66	3.66	
1’	104.6	105.8	4.44	4.44	7.0
2’	70.0	69.8	3.54	3.54	7.5, 10.5
3’	72.0	72.0	3.23	3.23	10.5
4’	70.8	70.5	3.38	3.39	10.0
5’	74.0	74.0	3.42	3.42	6.0, 9.0
6’	18.1	17.8	1.29	1.29	
3’-NMe_2	42.2	42.3	3.03	3.00	
6	39.1	38.9	1.61, 1.90	1.91	
7	70.0	69.9	3.83	3.83	
8	40.8	41.0	1.56	1.58	
8-Me	9.3	9.4	0.93	0.89	
9	79.9	79.9	3.90	3.90	7.5
10	137.1	136.9	-	-	
10-Me	12.4	12.4	1.65	1.65	
11	128.0	127.8	6.06	6.04	11.0
12	129.9	129.5	6.35	6.36	11.0, 14.5
13	131.7	131.6	5.81	5.80	
14	39.8	39.8	2.34	2.34	6.5
15	75.8	75.7	3.77	3.78	
16	42.6	42.9	1.52	1.52	
16-Me	7.4	7.4	0.95	0.96	
17	75.5	75.5	3.72	3.72	
18	36.1	36.1	1.48	1.47	
19	23.5	23.3	1.32, 1.34	1.32	
20	38.5	38.5	1.47	1.45	
21	71.1	70.9	3.55	3.55	
22	43.1	43.0	1.61, 1.67	1.61, 1.68	
23	77.5	77.4	4.23	4.23	7.0
24	139.4	138.5	-	-	
24-Me	12.2	12.4	1.64	1.64	
25	129.3	129.9	5.23	5.21	10.5

26	41.9	41.8	2.45	2.44	
1”	32.4	32.4	1.18, 1.79	1.19, 1.81	7.0
2”	26.6	30.6	1.37	1.38	
3”	35.8	34.9	1.31, 1.44	1.29	
4”	34.0	33.0	1.43, 1.49	1.28	
5”	29.2	14.4	1.29	0.88	
6”	11.5	-	0.87	-	
27	79.7	79.6	3.36	3.36	
28	74.2	74.0	3.47	3.47	
29	27.8	27.8	1.43, 1.68	1.42, 1.66	
30	36.1	36.0	1.43, 1.70	1.69	
31	70.1	69.9	3.83	3.83	
32	46.4	46.2	1.50, 1.57	1.51, 1.57	
33	66.8	66.7	4.06	4.07	
34	42.0	41.8	1.48, 1.50	1.49, 1.50	
35	72.9	72.8	3.74	3.74	
36	40.8	40.6	1.48	1.47	
36-Me	15.6	15.6	0.92	0.93	
37	29.8	29.6	1.28, 1.12	1.12, 1.56	
38	36.0	36.0	1.50	1.49	
39	76.3	76.3	3.72	3.72	
40	42.3	42.3	1.51	1.51	
40-Me	7.0	6.9	0.91	0.92	
41	75.9	75.9	3.71	3.71	
42	33.8	33.6	1.43, 1.62	1.43, 1.62	
43	22.9	22.9	1.43	1.45	
44	38.8	38.9	1.45, 1.46	1.45, 1.46	
45	70.6	70.5	3.67	3.67	
46	44.8	44.9	1.60, 1.65	1.60	
47	71.1	71.0	4.28	4.27	6.5, 13.0
48	136.7	136.8	5.80	5.77	5.0, 10.5
49	130.8	130.9	5.72	5.73	6.0, 15.5
50	72.8	72.8	5.43	5.42	6.0
51	20.8	20.8	1.33	1.35	

Supplementary Table 4: Crystallographic statistics

	apo-MccB	MccB + 1 Hex-CoA	MccB + 4 Hex-CoA
	**5ING**	**5INF**	**5INI**
**Data collection**	ALS BL821	ALS BL822	ALS BL821
Wavelength (Å)	0.9999	0.9998	1.000
Space group	$P2_12_12_1$	$P2_12_12_1$	$P2_12_12_1$
Cell dimensions			
a, *b*, *c* (Å)	120.56, 163.44, 186.55	110.072 165.453 190.261	110.978,161.526, 187.294
α, β, γ (°)	90, 90, 90	90, 90, 90	90, 90, 90
Resolution (Å)	58.12 - 2.45	42.22 - 2.75	48.44 - 2.85
R_{merge}	0.112 (0.966)	0.208 (0.837)	0.206 (0.731)
$I/\sigma I$	11.8 (1.6)	14.4 (3.5)	10 (3.6)
Completeness (%)	99 (97.3)	100 (99.9)	100 (99.4)
Multiplicity	6.1 (5.4)	7.4 (7.3)	6.9 (6.7)
CC(1/2)	(0.662)	(0.796)	(0.820)
**Refinement**			
Resolution (Å)	58.12 - 2.45	42.22 - 2.75	48.44 - 2.85
No. reflections	133794 (12965)	90300 (8650)	79173 (7792)
$R_{work/}$ R_{free}	0.195/0.223	0.184/0.242	0.173/0.230
No. atoms			
Protein	22424	21976	22284
Ligand/ion	N/A	55	220
Water	956	1091	392
B-factors			
Protein	48.57	27.71	24.59
Ligand/ion	N/A	52.88	54.39
Water	47.12	26.09	20.27
R.M.S deviations			
Bond lengths (Å)	0.007	0.013	0.007
Bond angles (º)	1.07	1.17	0.90

All structures were determined from a single crystal

*Highest resolution shell is shown in parenthesis.

Supplementary References

1. Quade, N., Huo, L., Rachid, A. Heinz, D.W. & Müller, R. Unusual carbon fixation gives rise to diverse polyketide extender units. *Nat. Chem. Biol.* **8**, 117-124 (2012)
2. Peter, D.M. *et al.* Screening and engineering the synthetic potential of carboxylating reductases from central metabolism and polyketide biosynthesis. *Angew. Chem., Int. Ed.* **54,** 13457-13461 (2015)
3. Zhang, L. *et al.* Rational control of polyketide extender units by structure-based engineering of crotonyl-CoA carboxylase/reductase in antimycin biosynthesis. *Angew. Chem., Int. Ed.* **54,** 13463-13465 (2015)
4. Laureti, L. *et al.* Identification of a bioactive 51-membered macrolide complex by activation of a silent polyketide synthase in Streptomyces ambofaciens. *Proc. Natl. Acad. Sci. U. S. A.* **108,** 6258-6263 (2011)
5. Kieser, T., Bibb, M. J., Buttner, M. J. Chater, K. F. & Hopwood, D. A. *Practical Streptomyces Genetics*, John Innes Foundation, Norwich (2000).
6. Osada, H., Koshino, H., Isono, K., Takahashi, H. & Kawanishi, G. Reveromycin A, a new antibiotic which inhibits the mitogenic activity of epidermal growth factor. *J. Antibiot. (Tokyo)* **44,** 259-261 (1991)
7. Komatsu, M., Uchiyama, T., Omura, S., Cane, D. E. & Ikeda, H. Genome-minimized Streptomyces host for the heterologous expression of secondary metabolism. *Proc. Natl. Acad. Sci. U. S. A.* **107,** 2646-2651 (2010)
8. Miyazawa, T *et al*. Identification of middle chain fatty acyl-CoA ligase responsible for the biosynthesis of 2-alkylmalonyl-CoAs for polyketide extender unit. *J. Biol. Chem.* **290,** 26994-27011 (2015)
9. Kindly provided by Dr Jean-Luc, Pernodet, Université Paris-Sud 11, Orsay, France.
10. Onaka, H., Taniguchi, S., Ikeda, H., Igarashi, Y. & Furumai, T. pTOYAMAcos, pTYM18, and pTYM19, actinomycete-Escherichia coli integrating vectors for heterologous gene expression. *J. Antibiot. (Tokyo)* **56,** 950-956 (2003)
11. Takahashi, S. *et al.* Biochemical characterization of a novel indole prenyltransferase from Streptomyces sp. SN-593. *J. Bacteriol.* **192,** 2839-2851 (2010)
12. Hong, H., Samborskyy, M., Lindner, F. & Leadlay, P.F. An amidinohydrolase provides the missing link in the biosynthesis of amino marginolactone antibiotics. *Angew. Chem., Int. Ed.* **55,** 1118-1123 (2016)
